# Intercellular Redistribution of cAMP Underlies Selective Suppression of Cancer Cell Growth by Connexin26

**DOI:** 10.1371/journal.pone.0082335

**Published:** 2013-12-03

**Authors:** Anjana Chandrasekhar, Edward A. Kalmykov, Srikanth R. Polusani, Sandra A. Mathis, Shoshanna N. Zucker, Bruce J. Nicholson

**Affiliations:** 1 Department of Biochemistry, University of Texas Health Science Center at San Antonio, San Antonio, Texas, United States of America; 2 Department of Pharmaceutical, Social and Administrative Sciences, D'Youville College School of Pharmacy,Buffalo, New York, United States of America; University of California Merced, United States of America

## Abstract

Connexins (Cx), which constitute gap junction intercellular channels in vertebrates, have been shown to suppress transformed cell growth and tumorigenesis, but the mechanism(s) still remain largely speculative. Here, we define the molecular basis by which Cx26, but less frequently Cx43 or Cx32, selectively confer growth suppression on cancer cells. Functional intercellular coupling is shown to be required, producing partial blocks of the cell cycle due to prolonged activation of several mitogenic kinases. PKA is both necessary and sufficient for the Cx26 induced growth inhibition in low serum and the absence of anchorage. Activation of PKA was not associated with elevated cAMP levels, but appeared to result from a redistribution of cAMP throughout the cell population, eliminating the cell cycle oscillations in cAMP required for efficient cell cycle progression. Cx43 and Cx32 fail to mediate this redistribution as, unlike Cx26, these channels are closed during the G2/M phase of the cell cycle when cAMP levels peak. Comparisons of tumor cell lines indicate that this is a general pattern, with growth suppression by connexins occurring whenever cAMP oscillates with the cell cycle, and the gap junction remain open throughout the cell cycle. Thus, gap junctional coupling, in the absence of any external signals, provides a general means to limit the mitotic rate of cell populations.

## Introduction

Gap junctions are arrays of intercellular channels that are the only mediators of direct intercellular exchange of small metabolites and signaling molecules in multicellular systems [1,2]. In vertebrates, these channels are composed of integral membrane proteins called connexins (Cx), with four transmembrane domains and cytoplasmic N and C –termini. Six connexins come together to form a hemichannel or connexon, and two such hemichannels from opposing cells dock to create the intercellular gap junction channel [3]. The integral, but specialized role of gap junctions in varied tissues is facilitated by the presence of at least 21 different connexin isoforms in humans, with distinct proteins that are classified according to their molecular weight [4], and a gene nomenclature described in [5]. The Cx43, Cx32 and Cx26 proteins studied here are encoded by the *GJA1*, *GJB1* and *GJB2* genes, respectively. 

Almost since their discovery, gap junctions have been implicated as tumor suppressors in several tissues [6]. This has been confirmed in genetic screens of numerous tumor types including breast carcinoma [7], prostate cancer [8], and melanoma [9]. A multitude of tumor types and transformed cell lines demonstrate decreased connexin protein expression and/or gap junction functionality (reviewed in 10). Furthermore, there are several documented cases where the exogenous expression of connexins in a transformed cell line can dramatically suppress its transformed properties and its ability to form tumors in nude mice [Cx43 in C6 glioma cells [11]; Cx43 in 10T1/2 embryonic mesenchymal cells [12]; Cx43 and Cx32 in LNCaP prostate cancer cells [13]; Cx26 in HeLa cervical cancer cells [14]; Cx26 and Cx43 in MDA-MB-231 breast cancer cells [15,16], and; Cx32 in SKHep1 hepatoma cells [17]. The latter is also consistent with an increase in hepatic tumorigenesis observed in Cx32-/- mice [8,18].

Growth suppression of transformed cells by Cx expression has been linked to regulation of pro- and anti-apoptotic proteins (e.g. Bcl-2) [19,20] or changes in cell cycle proteins such as NOV (CCN3) [21], Skp2 [22] and p21 [23,24]. However, establishing a direct connection between any of these events and the exchange of signaling molecules between cells through gap junctions has proven elusive. Progress in this regard has been restricted by limited information on both the permeability properties of connexins, and the spatio-temporal distribution of low molecular weight metabolite concentrations in multicellular populations. In some cases, connexins have been proposed to suppress growth even in the absence of demonstrable gap junction channel activity [15,21,24,25,]. This could occur through interactions with other proteins known to bind to connexins (reviewed by [26]), through their function as hemichannels, which can contribute to increased cell death [27], or even through mis-localization of parts of the protein (25). However, definitive links between any of these processes and anti-oncogenic factors remain to be established.

While the role of cell coupling, compared to other connexin functions, is still a subject for debate in tumor suppression, the link between gap junction coupling and mitogenesis has been established in several non-pathogenic conditions. Several growth factors, such as EGF [28] and PDGF [12] have been shown to induce transient uncoupling of cells as part of the immediate early response that precedes initiation of mitosis. Oncogenes like *v-src* have been shown to have similar, but more long-lasting effects on coupling [29]. *In vivo*, as part of the regenerative response following partial hepatectomy, an acute loss of gap junctions between hepatocytes is seen to immediately precede the first wave of mitosis [30]. However, as has been the case in tumors, the molecular basis of the mitogenic inhibition associated with cell coupling remains to be resolved. In this study, we sought to define the molecular mechanism of growth suppression by connexins in HeLa cells, a very well characterized transformed cervical cancer cell line, and test the generalization of such mechanisms with other cell types. Our results demonstrate that Cx26 specifically allows the passage of cAMP between cells, leveling out the peaks and troughs of cAMP that occur during the cell cycle and, via PKA activation, causing delays of the cell cycle. The effect is connexin specific, as other connexins, like Cx43 and 32, close during the cell cycle at exactly the time when cAMP levels accumulate. Examination of several tumor cell lines indicates a generality to this effect as long as cAMP oscillations are seen and the channels remain open throughout the cell cycle. 

## Results

### Cx26, but not Cx32 or 43, inhibit transformed growth of HeLa cells

**Figure 1:**
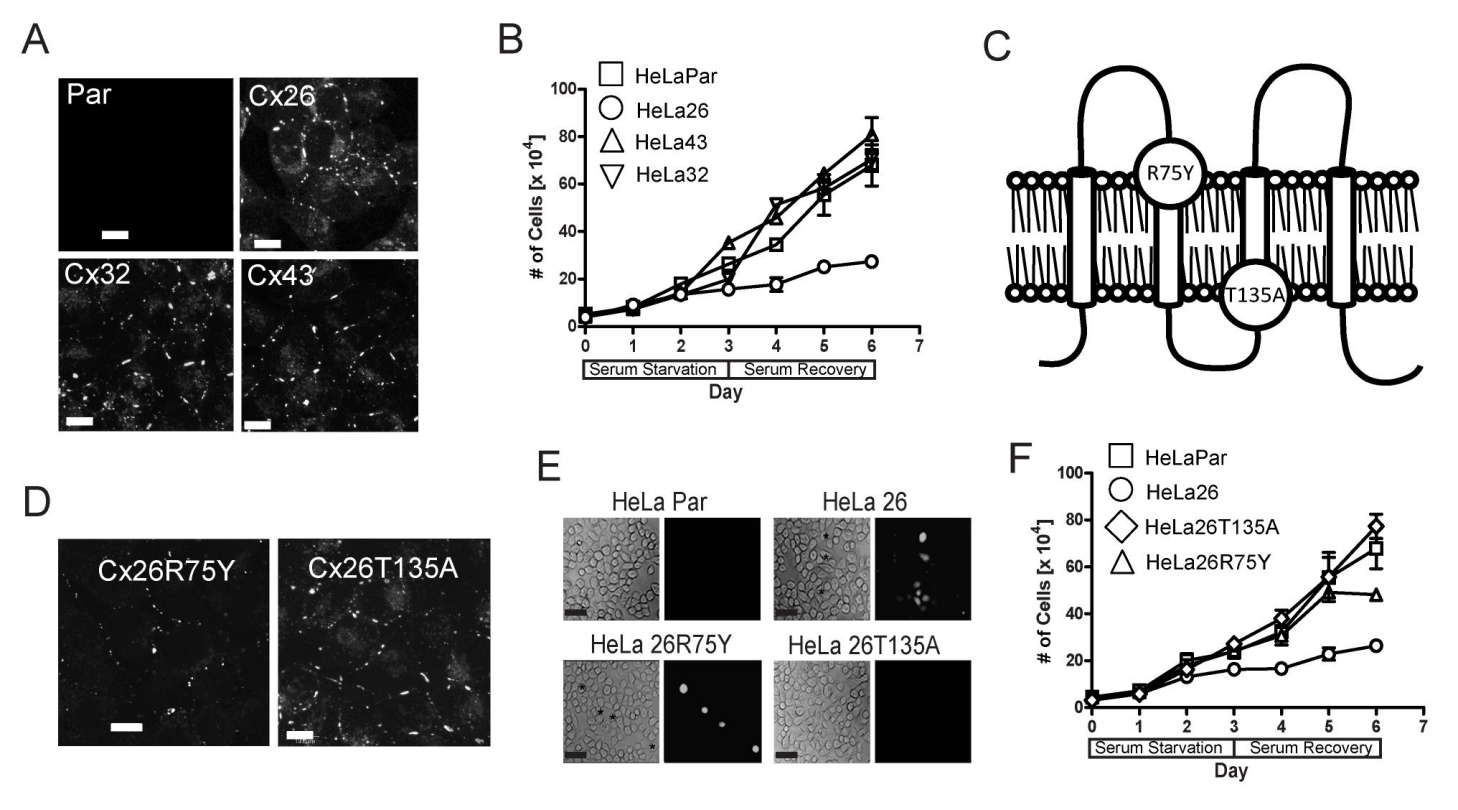
Characterization and growth behavior of HeLa cells expressing different w.t. and mutant connexins. (A) Immunofluorescence staining, using the appropriate anti-Cx antibody, shows characteristic expression of connexins in plaques between cells. HeLaPar tested negative with all the three anti-Cx antibodies (anti-Cx26 antibody results are shown here) (scale bars, 10μm). (B) Growth of HeLaPar (squares) with pools of HeLa clones transfected with Cx26 (circles), Cx32 (inverted triangle) or Cx43 (upright triangle), subjected to 3 days of serum starvation (days 0-3) followed by 3 days in 1% serum (days 3-6), reveals selective growth suppression by Cx26. Representative data from one of three experiments is shown, with points representing the mean ± SEM of triplicate platings. (C) Topology diagram of Cx26, with extracellular surface at the top, depicts the location of two point mutants used in this study that disrupt different aspects of channel function (R75Y and T135A). (D) Immunoflourescence staining with an anti-Cx26 antibody shows that both mutant connexins form plaques at the cell surface, with R75Y having slightly smaller, less frequent plaques, and T135A having larger and more frequent plaques than w.t. Cx26 (panel A) (scale bars, 10μm). (E) Hemichannel function was assayed by LY dye uptake in a dye dropping assay (Scout et al., 2002). The left panel shows the phase contrast image (asterisks indicate cells taking up the dye) and the right panel shows the fluorescent image (scale bars, 20μm). Note that some dye-positive cells appear to have received dye through intercellular transfer from the original cell that took up dye in the HeLa26 cultures. (F) Comparison of the growth in low serum (c.f. panel B) of mutant Cx26 expressing cells (Cx26T135A - diamonds, Cx26R75Y – triangles) with those expressing w.t. HeLa26 (circles) and HeLaPar cells (squares) demonstrates that the functional intercellular channels of w.t. Cx26 are required for growth suppression. Representative data from one of three experiments is shown, with points representing the mean ± SEM of triplicate platings.

HeLa cells are a broadly studied cervical cancer cell line, which lack endogenous gap junctions, but allow robust expression, appropriate trafficking, and processing of exogenously expressed connexins. We utilized this system to characterize connexin growth properties by preparing pooled clones of HeLa cells expressing Cx43 (HeLa43), Cx32 (HeLa32), and Cx26 (HeLa26). Each of the HeLa transfectants express connexins at similar levels, and according to the predicted MW on Western blots (Figure S1). They form typical gap junction plaques at the cell-cell interface (Figure 1A), and show similar rates of transfer of pre-loaded calcein dye to surrounding unlabeled cells, with ~95% of loaded cells being coupled to at least one neighbor, and 73-83 % of first order neighbors receiving dye (Table 1).

**Table 1 pone-0082335-t001:** Functional Analysis of Gap Junctions and Hemichannels.

	**Gap Junction**		**Hemichannel**
**HeLa Cell Type**	**% Coupled^a^**	**% First Order^b^ Coupling**	**% Cells Filling with Extracellular Dye^c^**
**Parental**	0	0	0
**Cx 43**	95 ± 1.32	73 ± 10.43	NT
**Cx 32**	96 ± 2.01	80 ± 9.53	NT
**Cx 26**	93 ± 0.67	83 ± 8.28	3 ± 1.7
**Cx26 R75Y**	0	0	6 ± 1.2
**Cx 26T135A**	0	0	0

^a^ the percentage of preloaded cells passing dye to at least one receiver cell.

^b^ the percentage of first-order cells (i.e. those immediately adjacent to the donor cell) receiving dye after 6 hrs of plating.

^c^ the percentage of cells showing dye uptake of Lucifer Yellow (LY) (see Figure 1E). Results do not include cells receiving LY secondarily through coupling (i.e. Cx26).

NT: Not tested

The effect of connexin expression on the transformed growth characteristics of HeLa cells was assessed under low (1%) serum conditions and on poly (2-hydroxyethyl methacrylate) (polyHEMA) coated plates that do not support cell-substrate adhesion [31]. Despite the similar levels of coupling in all connexin transfectants, we found that only HeLa26 showed growth retardation, consistent with previous studies (Mesnil et al., 1995). HeLa26 show 3-fold longer doubling times in low serum conditions (Figure 1B), and ≥2-fold slower anchorage independent growth (Figure S2) compared to parental HeLa cells (HeLa Par) or other connexin transfectants, as demonstrated in three independent HeLa26 clones. All data shown here was obtained from pools of clones mixed in equal ratios for each experiment. 

### Intercellular communication by Cx26 is necessary for growth suppression

The specific function of Cx26 responsible for growth suppression was assessed using Cx26 point mutations that ablated channel function. The Cx26T135A mutation at the cytoplasmic end of the third transmembrane domain (Figure 1C) prevents opening of either gap junction channels or hemichannels [32]. The Cx26R75Y mutation in the first extracellular loop (Figure 1C) prevents gap junction channel formation, but leaves hemichannel function largely unchanged [33]. Gap junction and hemichannel properties of all Cx26 constructs, as well as Cx43 and 32, were confirmed using Lucifer Yellow transfer between cells, or uptake from the media following mechanical stimulation (Figure 1E), respectively (summarized in [Table pone-0082335-t001]). Both Cx26 mutants form plaques in HeLa cells (Figure 1D), documenting that these mutations still support assembly of gap junction structures [32]. Plaques appeared to be more numerous in the case of HeLa26T135A, and smaller in the case of HeLa26R75Y (c.f. Figures 1A and D), consistent with previous indications that Cx26R75Y may reduce the stability and size of gap junction plaques [34]. 

Unlike wild-type (w.t.) Cx26, neither of the Cx26 mutants caused growth suppression of HeLa cells, either in low serum (Figure 1F), or under anchorage-independent conditions (Figure S2). Thus, intercellular communication is required for growth retardation by Cx26. While the hemichannel function of HeLa26R75Y did reduce saturation density, suggesting a partial role for hemichannels in the degree of cell packing, the effect was by a moderate 20%, in comparison to w.t. Cx26, which reduced saturation density by 60%. Although intercellular coupling was clearly necessary for connexin mediated reversal of the transformed growth characteristics, coupling through other connexins (Cx43 and Cx32) was ineffective, indicating a unique role for Cx26 channels. 

### Growth effects of Cx26 gap junctions are mediated by reversible cell cycle blocks

**Figure 2:**
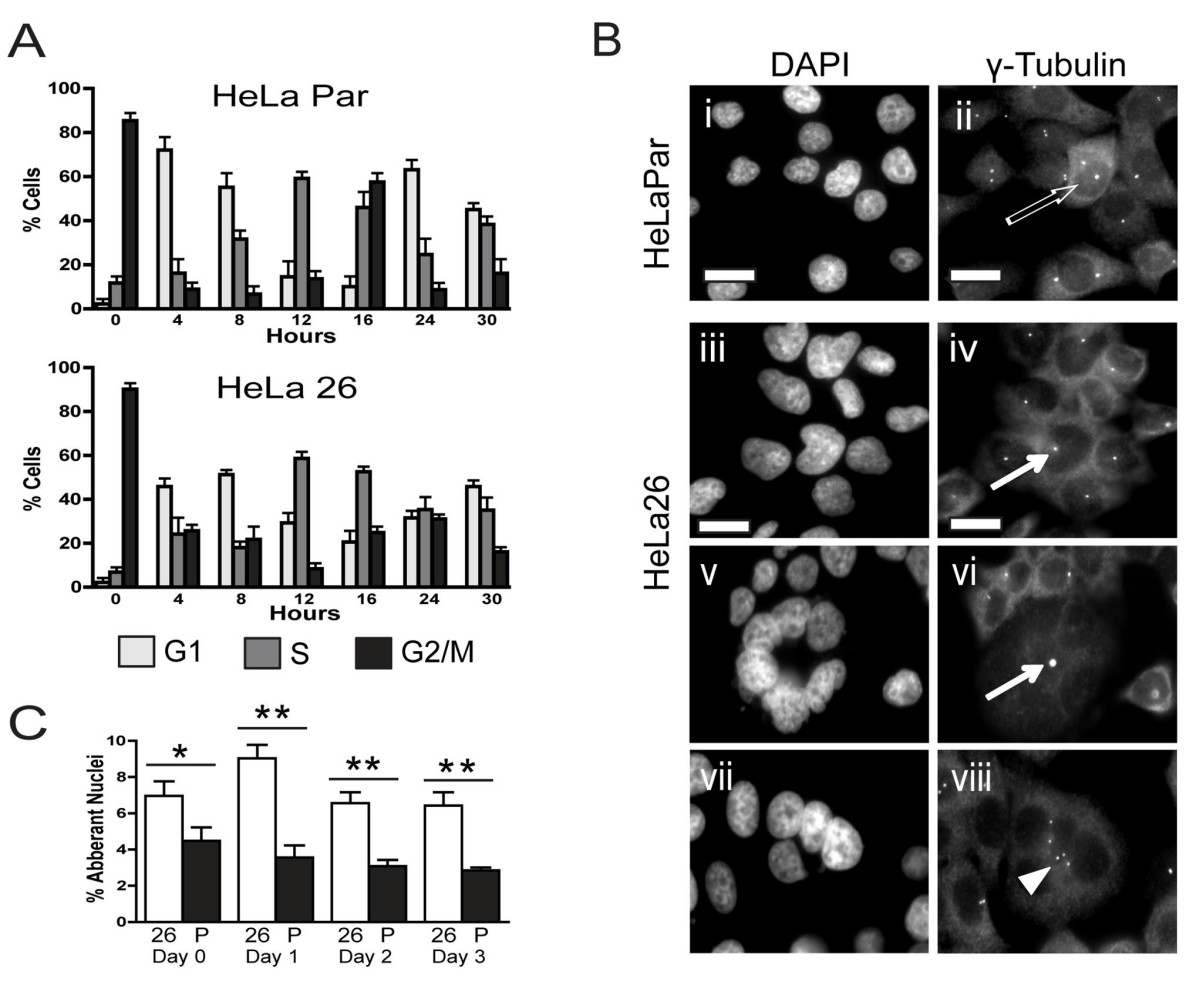
Effects of Cx26 coupling on the cell cycle. (A) The cell cycle distribution of HeLaPar (top panel) and HeLa26 (bottom panel) was analyzed by FACS [separating cells into G1 (light grey bars), S (dark grey bars) and G2-M (black bars) phases] at the indicated times after 1% serum addition following serum starvation. HeLa26 cells show a slower progression through the cell cycle (e.g. time to return to G2: >24 hours compared to 16 hours for HeLaPar), as well as a notable loss of synchronization. Data are means ± SEM of three independent experiments. (B) Staining of the nucleus with DAPI (left column) reveals a much higher frequency of multilobed nuclei in Cx26 expressing cells (10-14% - panels iii, v and vii) than HeLaPar cells (2 -5 % - panel i). Co-staining the same cells for gamma-tubulin (right column) shows either one (quiescent cells) or two (dividing cells) centrosomes in HeLaPar cells (panel ii). In contrast, HeLa26 cells (panels iv, vi and viii) often share a single centrosome among multiple nuclei (arrows), or have a cluster of multiple centrioles (arrowhead) (scale bars, 10μm). (C) The percent of multi-lobed cells determined at different days after addition of 1% serum, is consistently higher in HeLa26 (filled bars) than HeLaPar (open bars), with the maximum difference at 1 day. Data is mean ± SEM. * denotes significance to p value <0.05. ** denotes significance to p values <0.005.

As a first step in identifying the mechanism by which Cx26 gap junction coupling suppresses transformed growth of HeLa cells, we asked if this was a consequence of increased cell death, or reduced cell division. HeLa26 cells showed no increase in TUNEL staining, or caspase-3 activity (not shown), indicating that Cx26 did not increase apoptosis in HeLa cells (Table S1). There was some increase in Annexin V and Hoechst labeling of HeLa26 cells, but these cells did not show morphological features characteristic of cell death by apoptosis, necrosis or autophagy. Instead, they showed features consistent with cells that may have been blocked in cytokinesis (see below), which typically show greater membrane permeability. FACS analysis of HeLa Par and Cx26 transfectants following release into 1% serum from thymidine/nocodazole block (G2 arrest), revealed a prolonged exit from mitosis in HeLa26 cells, with apparent delays in all phases of the cell cycle. (Figure 2A). HeLa43, HeLa32, and the two mutant Cx26 HeLa transfectants show cell cycle distributions similar to HeLa Par (data not shown). Similar studies comparing growth after release from G1 block induced by double thymidine block yielded similar results, with exit from G1/S being notably delayed in HeLa26 cells compared to HeLa43, HeLa32, and the mutant Cx26 HeLa transfectants (data not shown).

 Hoechst or DAPI staining in 1% serum revealed a significant accumulation of polymorphic nuclei, or multi-nucleated cells in HeLa26 (≥10% 24 hrs after serum addition) compared to that seen in HeLa Par (<4%) (Figure 2B). These cells are significantly larger and more flattened than those with single nuclei, and appear consistent with failure to successfully complete M phase and/or cytokinesis. These nuclear abnormalities are associated with either single (arrows in Figure 2B, iv and vi), or multiple (arrowhead in Figure 2B viii) centrosomes within a single cell, shared by multiple nuclei, or nuclear lobes. This is in contrast to the usual two centrosomes associated with dividing HeLa Par cells (open arrow in Figure 2B ii), and suggests that the HeLa26 cells have problems with the regulation of centrosome duplication. Intact nuclear membranes, the lack of condensed chromatin, and the absence of TUNEL labeling in HeLa26 (Table S1), indicate that the cells do not enter apoptosis, but eventually exit mitosis. Consistent with this, while the multi-lobed or multi-nucleated cells are always more abundant in HeLa26 cultures, they do not accumulate over time (Figure 2C).

### Cx26 gap junction coupling affects levels and activity of several mitogenic signaling molecules

**Figure 3:**
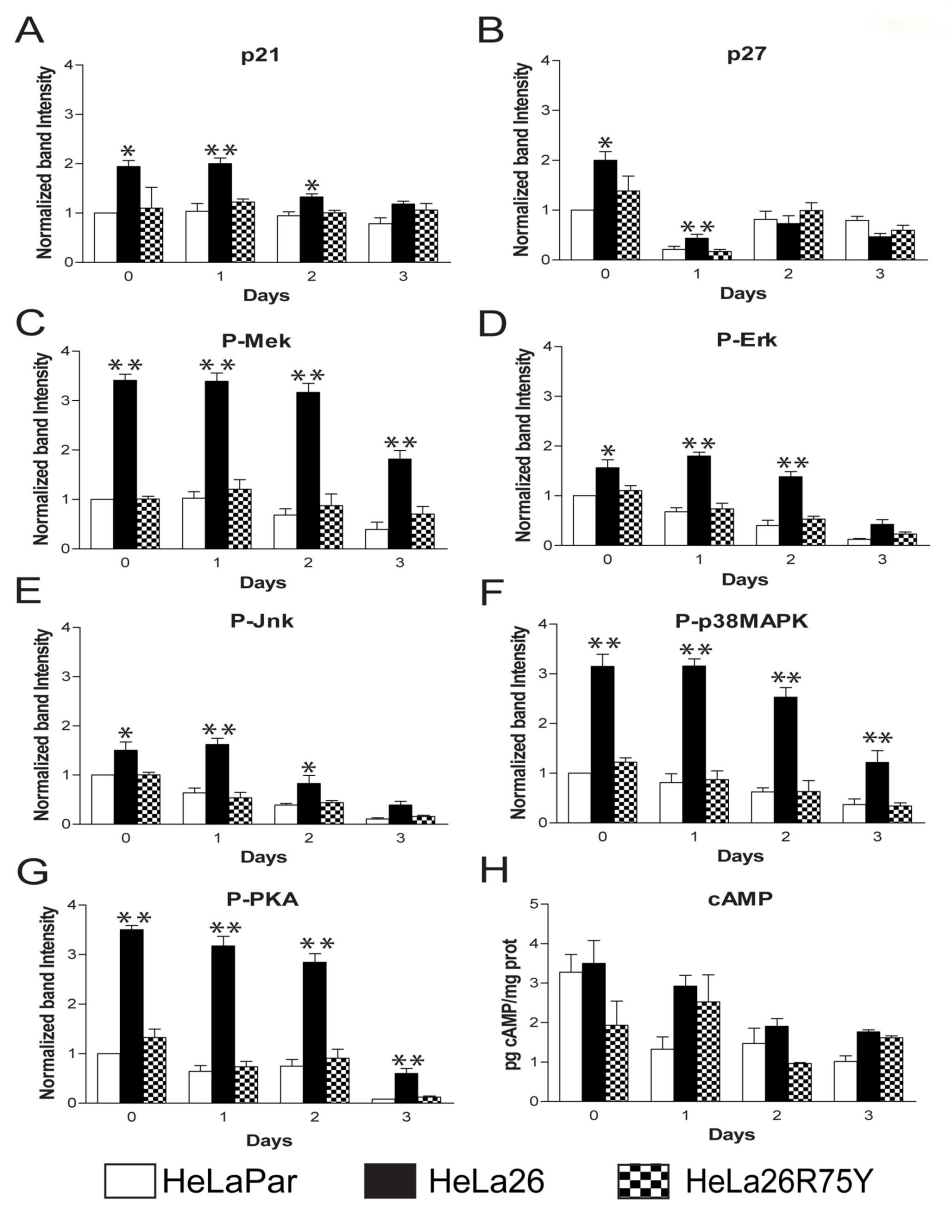
Effect of Cx26 on cdk inhibitor protein levels and mitogenic kinase activities. (A-G) Protein levels in HeLaPar (open bars), HeLa26 (closed bars) and HeLa26R75Y (checkered bars) were quantified from digitized images of Western blots (Figure 2, Supplementary Data) immediately after serum starvation (day 0), and daily after 1% serum addition. Levels for each protein were normalized to that of HeLaPar cells at day 0. Actin served as the internal loading control. Levels of the cdk inhibitors, p21 (A) and p27 (B), and the ratio of phosphorylated (activated) to total levels of the MAPK family members and their regulators, MEK (C), ERK (D), JNK (E) p38MAPK (F) and the catalytic subunit of PKA (G) were each higher in HeLa26. This elevation, evident during serum starvation, persisted from 1 - 3 days, depending on the protein. (H) cAMP levels were assessed by ELISA in HeLaPar (open bar), HeLa26 (closed bar) and HeLa26T135A (checkered bar). Unlike PKA activity, cAMP levels do not show consistent increases in HeLa26 compared to the other cell types. HeLa26R75Y cells show cAMP levels similar to HeLa26T135A. Data are the means ± SEM from at least three independent experiments. Asterisks indicate cases where activity in HeLa26 is significantly higher (* = p < 0.05; ** = p < 0.005) than BOTH HeLaPar and HeLa26R75Y or T135A cells.

Consistent with the observed effects of Cx26 on the cell cycle of HeLa cells, the activation state of several mitogenic regulators was found to be chronically increased in HeLa26 compared to HeLa Par cells and other Cx transfectants (Cx26R75Y shown as an example) during growth in 1% serum (Figure 3 and Figure S3). Specifically, the levels of the CDK inhibitors, p21 and p27, which directly regulate G1 (p21 and p27) and G2 (p27) progression, were 1.5- to 2-fold higher in HeLa26 cells during serum starvation (0 time point), and remained higher for 48 or 24 hours after 1% serum addition, respectively. 

Several members of the mitogen activated protein kinase (MAPK) family, known to regulate p21 and p27 levels (Erk 1/2, p38 and JNK) (Figure 3 C-E), showed persistent increases (2- to 3-fold) in HeLa26. Two kinases further upstream, MEK and the protein kinase A (PKA) catalytic subunit alpha (Figure 3 F and G), showed even greater prolonged increases in activity in HeLa26 (3- to 5-fold for over 72 hours following serum addition). These increases were not seen in all mitogenic pathways as no changes in Akt or p70 S6 kinase were observed. 

While prolonged activation of MAPKs is associated with decreased cell division [35,36], their acute activation is typically pro-mitogenic. Consistent with this, in the first 2 hours following 1% serum stimulation, HeLa Par cells showed a transient activation of Erk and JNK and a decrease in p38, followed by resumption of the basal state. In contrast, HeLa26 did not show these transient changes in activity (Figure S4), but only a slower onset activation starting ~2 hours after serum addition.

### Activation of PKA is both necessary and sufficient for growth suppression by Cx26

**Figure 4:**
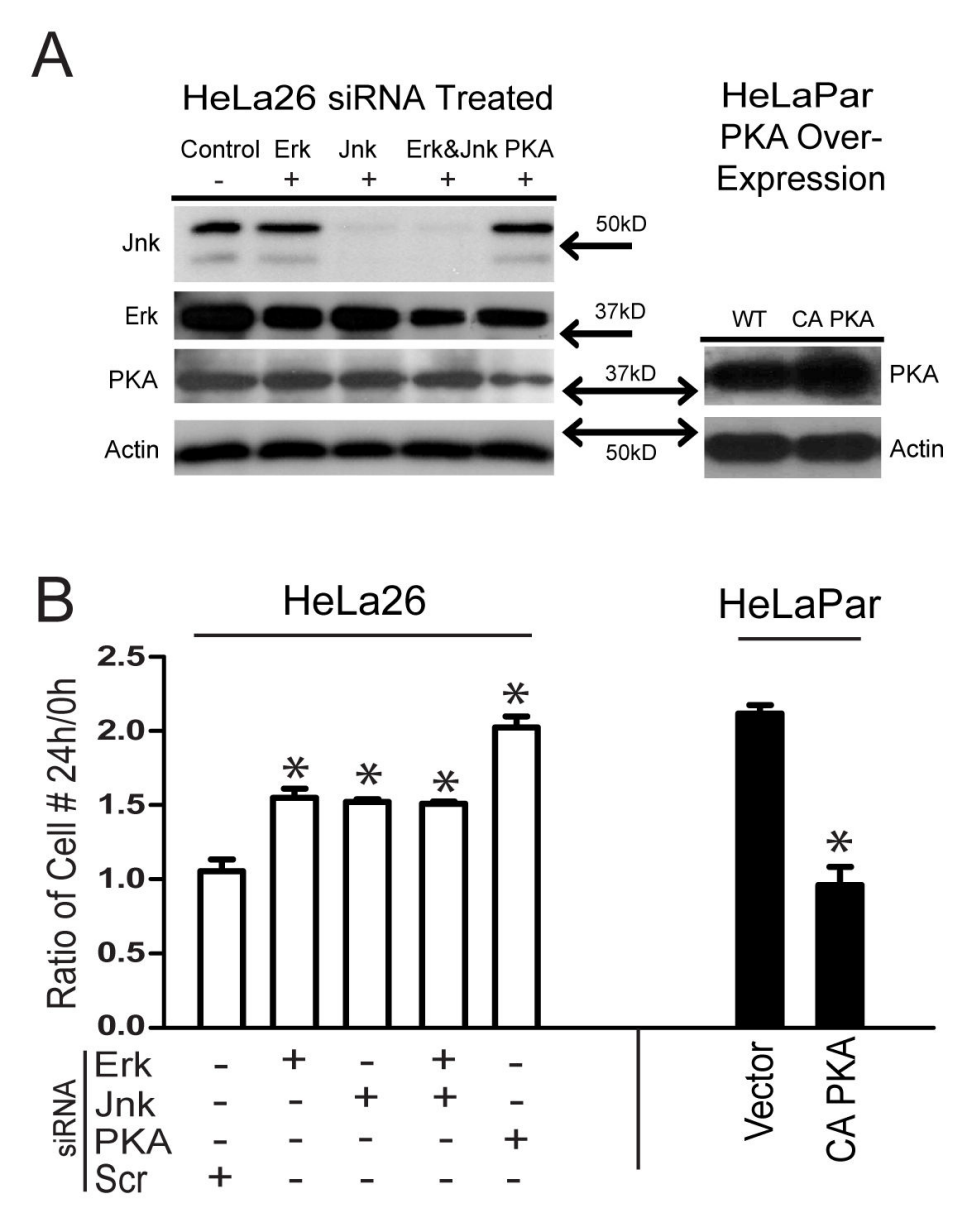
Effects on cell growth by inhibition of kinases with siRNA, and expression of a constitutively active form of PKA. (A) Lysates of HeLa26 (left panel), treated with random sequence siRNA (control), or siRNAs specific for the kinases indicated, were prepared 24h after serum addition and probed with antibodies against total protein as indicated to the left of each blot. Levels of all kinases were reduced, with JNK decreases being most dramatic. Lysates of HeLaPar cells (right panel) transfected with a CA-PKA (constitutively active) construct showed much higher expression of PKA than untransfected controls. Actin served as a loading control. (B) Growth of the different siRNA treated HeLa26 cells (open bars), or HeLaPar cells with and without constitutively active PKA expression (closed bars) are compared as a ratio of cell number 24h after, and at the time of, serum addition. The role of constitutive activation of each of the mitogenic kinases in growth inhibition of HeLa26 is evident in the reversal of Cx26 induced growth suppression by their respective siRNAs. This is most dramatic in the case of PKA, where growth returns to the same levels as HeLaPar cells. Growth suppression of HeLaPar cells, to the same degree as seen in HeLa26, by CA-PKA expression indicates that PKA alone can mediate this effect. Significant differences in growth are indicated by asterisks (p<0.05).

The functional contribution of the elevation of these kinase**s** to Cx26 growth suppression was assessed by siRNA knockdown studies of Erk, JNK and the PKA catalytic subunit alpha. siRNAs added during initial serum starvation, 24 hours before the addition of 1% serum, caused a 5-fold reduction in JNK and a 2-fold reduction of Erk and PKA (Figure 4A). Higher siRNA levels were not used to avoid toxic effects on the cells, as each of these signaling pathways is also required for basal cell division.

During the first 24 hours in 1% serum, HeLa Par cells doubled in number, while HeLa26 cells showed no significant growth (Figure 4B), consistent with the delay in cell cycle progression observed in the FACS analysis in Figure 2A. Treatment with siRNA of random sequence had no effect on this growth pattern. Erk and JNK siRNAs individually, or together, caused a partial loss of growth inhibition in HeLa26 cells, but siRNA to PKA caused a complete reversion to the growth pattern of HeLa Par cells. Furthermore, over-expression of the PKA catalytic subunit alpha in HeLa cells (Figure 4A, right panel) suppressed their growth to the same rate as that of HeLa26 cells (Figure 4B). Thus, while activation of other downstream kinases contributes to the regulation of HeLa cell growth, PKA activation appears to be the predominant influencing factor, being both necessary and sufficient for the effects of Cx26 on HeLa growth in low serum. 

### Activation of PKA by Cx26 gap junctional coupling is caused by redistribution of cAMP, not by elevation in its level

The primary role of PKA demonstrated above strongly implicates cAMP as a likely growth regulatory signal. However, comparisons of global cAMP levels in HeLa26, HeLa Par and HeLa26R75Y cultures (Figure 3H) revealed no consistent differences between the cell lines over three days of growth in 1% serum. However, cAMP has been shown to be permeable through Cx26 gap junction channels between HeLa cells [37,38]. Since functional intercellular channels are required for PKA activation and growth suppression, this led us to investigate if a diffusion of cAMP from cells with high concentrations to cells with lower concentrations could result in a larger fraction of cells with activated PKA, without any requirement for additional cAMP production.

**Figure 5:**
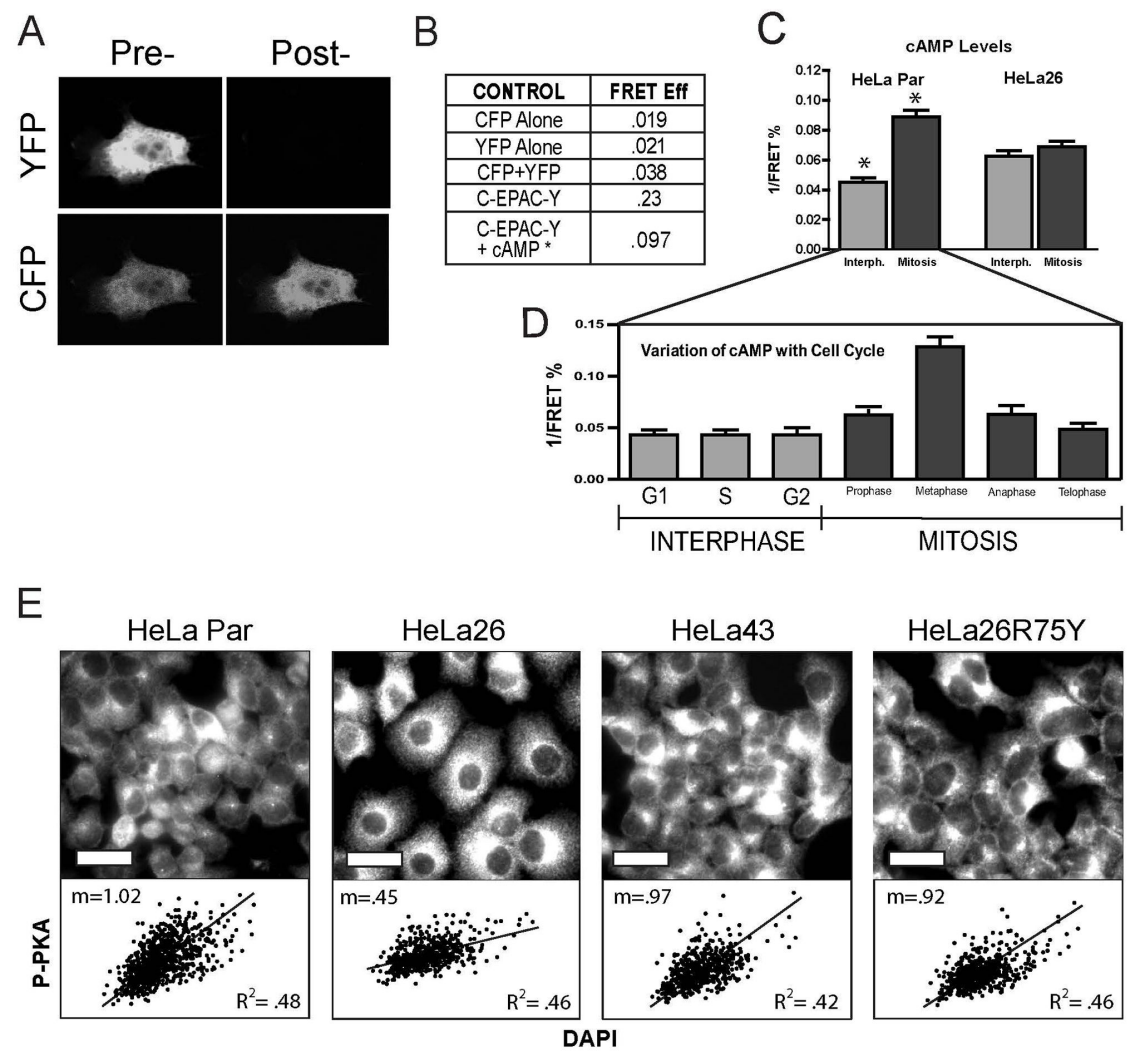
Cx26 expression in HeLa cells causes a redistribution of cAMP from mitotic to interphase cells, resulting in a more uniform activation of PKA. (A) Representation of acceptor photobleaching FRET, showing an increase in donor (CFP) fluorescence following complete acceptor (YFP) photobleaching. (B) FRET efficiency is minimal when CFP and YFP are expressed separately, or together, but not linked through a common carrier. FRET efficiency of the doubly labeled EPAC construct increases by almost 10 fold, but drops to only 3-4 fold above background when cAMP levels are increased. (C) FRET measurements 1 day after serum addition from individual cells in either interphase or mitosis show that the changes in cAMP levels with the cell cycle seen in HeLa cells is eliminated upon expression of Cx26. (D) FRET analysis of a population of HeLaPar cells synchronized in G1, shows the oscillatory pattern of cAMP with the cell cycle, with levels remaining low throughout interphase, and peaking during the metaphase portion of mitosis. Scale bar represent ~40µM. A minimum of 25 cells was averaged for each cell type and cell cycle stage for FRET. (E) Immunocytochemistry of active phospho-PKA (catalytic subunit alpha) 1 day after 1% serum addition (top row) reveals a heterogeneous staining pattern in HeLaPar cells and cells expressing Cx43 or the gap junction channel deficient Cx26R75Y mutant. Conversely, the p-PKA levels in HeLa26 are higher and more uniform across all cells. Scoring individual cells for net p-PKA signal and DNA content as assessed by DAPI staining reveals a correlation (lower graphs), with p-PKA levels being low in G1/S (lower DNA content) and high in G2/M (higher DNA content). This difference is dramatically reduced in HeLa26 cultures.

In order to directly visualize cAMP levels spatially, a FRET reporter employing EPAC as the cAMP sensing domain sandwiched between CFP and YFP fluorophores was introduced into the cells by transfection. Bleaching of the YFP (the acceptor) fluorphore resulted in an increase in CFP (donor) fluorescence (23%) (Figure 5A) that was not seen when CFP and YFP were expressed alone (2%) or together, but on different molecules (~4%). Elevation of cAMP by 8-Br cAMP (cAMP agonist), forskolin (adenyl cyclase activator) and 3-isobutyl-1-methylxanthine (phosphodiesterase inhibitor) caused a loss of FRET to <10%, presumably due to extension of the EPAC molecule on cAMP binding (data summarized in Figure 5B). FRET readings from individual cells in non-synchronized HeLa Par and HeLa26 cultures imaged in a confocal microscope were correlated with DNA content and nuclear morphology. The cAMP levels in HeLa Par cells increased ~ 2-fold from interphase to mitosis. This difference was almost completely lost in HeLa26 cells, due to increased interphase levels and decreased levels in mitotic cells (Figure 5C). Tracking cAMP levels throughout a normal cell cycle in HeLa par cells synchronized by double thymidine bloc, confirmed that cAMP levels remain low during G1 and S, rise in G2, peak in metaphase, and then decline in the latter part of mitosis (Figure 5D). These “peaks and valleys” of cAMP appear to be eliminated in the HeLa26 cells due to the diffusion of cAMP from the relatively few M phase cells, to the majority of G1/S phase cells. The resulting increase in cAMP in the G1/S phase cells induces activation of PKA, which is growth inhibitory at this stage of the cell cycle [39]. This explains the results in Figure 4B, where knock-down of PKA (particularly during G1/S phase) can eliminate the Cx26 growth suppressive effects of Cx26, while its activation can mimic it.

To confirm if this redistribution of cAMP was consistent with the overall increase in PKA phosphorylation at the activating Thr197 site [40] we had observed (Figure 3G), we also examined the spatial patterns of p-PKA staining in HeLa Par, 26, 43 and 26R75Y cultures (Figure 5E). The staining of individual cells was highly heterogeneous in all the cultures showing rapid growth in low serum (HeLa Par, Cx43 and Cx26R75Y). Co-staining with DAPI revealed a clear correlation between the intensity of P-PKA labeling of each cell with its DNA content (Figure 5E - plots below each immunofluorescent image), with the higher levels of P-PKA after DNA replication being consistent with the observed peak in cAMP levels in M phase shown in Figure 5B. HeLa26 cells presented an exception to this pattern in that the staining of cells was more uniform. Not surprisingly, the correlation of P-PKA intensity with DNA content was also dramatically reduced, consistent with the minimal differences we see in cAMP levels between M phase and S phase cells in these cultures (Figure 5C). These results are only readily explained by a redistribution of cAMP through Cx26 channels, from cells with higher concentrations (M phase), to cells with lower concentrations (G1/S phase). This apparently results in a much higher fraction of cells reaching cAMP levels sufficient to induce phosphorylation of PKA, leading to its activation and an overall upregulation of PKA activity in the culture without any actual increase in overall cAMP levels.

### Cx43 and Cx32 channels were ineffective in suppression of growth as these channels close during the cell cycle

**Figure 6:**
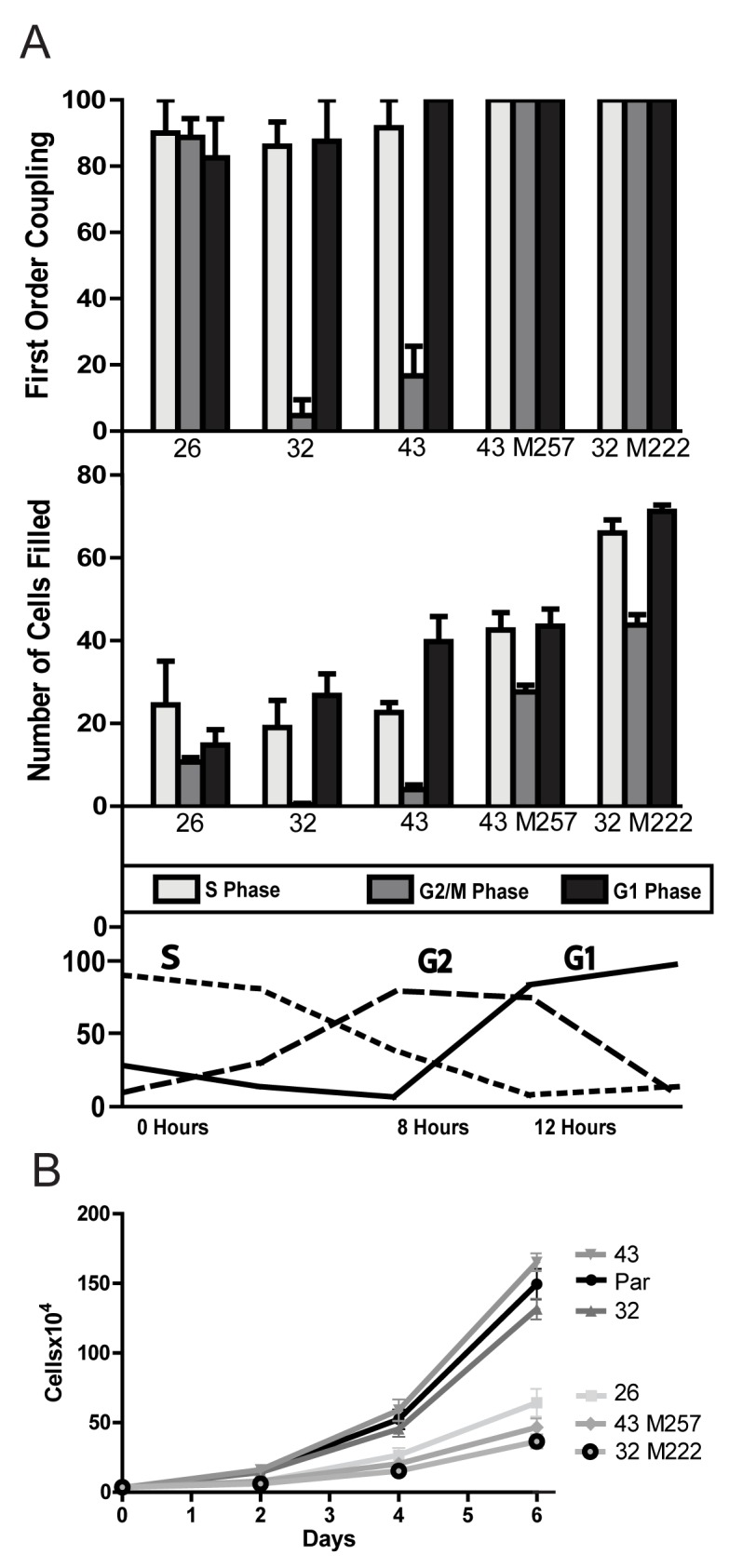
Changes in coupling mediated by different connexins during the cell cycle. (A) The fraction of cells in each phase of the cell cycle was determined by FACS at different times after release from double thymidine block (lower panel). Cell coupling after microinjection of Alexa 350 was measured as either the percent of first-order cells filled with dye (top) or total number of cells filled (middle) at S phase (0-4 hours – light grey bars), G2/M phase (8-10 hours – dark grey bars) or G1 phase (14 hours – black bars). Both Cx32 and Cx43 expressing cells showed dramatically reduced coupling during mitosis (G2/M), while HeLa26 cells remain coupled. Removal of the Cx43 or Cx32 C-terminal domains (Cx43M257 and Cx32M222, respectively) resulted in de-regulation of channel activity throughout the cell cycle, similar to Cx26. (B) Expression of CTD-deleted Cx43 or Cx32 resulted in reduced HeLa cell growth in low serum to levels comparable to those of cells expressing w.t. Cx26.

 Because HeLa43 and HeLa32 cells show robust intercellular coupling, and given prior evidence that cAMP can pass effectively through both Cx43 and Cx26 channels in these cells [37], we sought to determine why Cx43 and 32 were ineffective at suppressing growth. Since cAMP levels only increase during M phase, where it has been reported that gap junctional coupling can decrease, we examined if these three connexins are regulated differentially during the cell cycle. Cells were synchronized in S phase by double thymidine block in normal (10%) serum. Unlike the behavior in low serum (Figure 1), all HeLa cell lines grew at similar rates in normal serum. The degree of synchrony, as well as cell cycle progression after release from block, was found to be the same for all three connexin transfectants in normal serum conditions. Based on flow cytometry analysis, the cells reached G2 by 6-8 hours after thymidine release, entered M phase after 9 hours, and re-entered G1 by ~12 hours (Figure 6A lower panel). Cell coupling, assayed by microinjection of Alexa 350 fluorescent dye, decreased dramatically in HeLa43 and HeLa32 cells during G2, and reached a minimum during M phase (Figure 6A – upper two panels). In contrast, no reduction in first order coupling was seen at any cell cycle stage in HeLa26 cells (Figure 6A – upper panel), and only a modest reduction in total cells filled was observed during M phase (Figure 6A – middle panel), probably reflecting reduced cell contact. Thus, of the w.t. connexins tested, only Cx26 channels remain open at the time in the cell cycle when cAMP levels reach a maximum, and retain the capacity to redistribute it to surrounding cells.

 Consistent with the concentration of regulatory elements, such as phosphorylation sites, in the C-terminal domains of connexins, when C-terminally truncated forms of both Cx43 and 32 were transfected into HeLa cells, the cells remained coupled throughout the cell cycle, as seen in Cx26 (Figure 6A). When these truncated forms of Cx43 and Cx32 were expressed in HeLa cells, they also induced growth suppression of HeLa cells under low serum growth conditions, in contrast to their full length counterparts (Figure 6B). Thus, we can conclude that the specificity of Cx26 for growth suppression is due to its ability to remain coupled at critical times during the cell cycle, where redistribution of signals like cAMP can result in suppression of growth.

### Generality of growth suppression in different cancer cell lines

**Figure 7:**
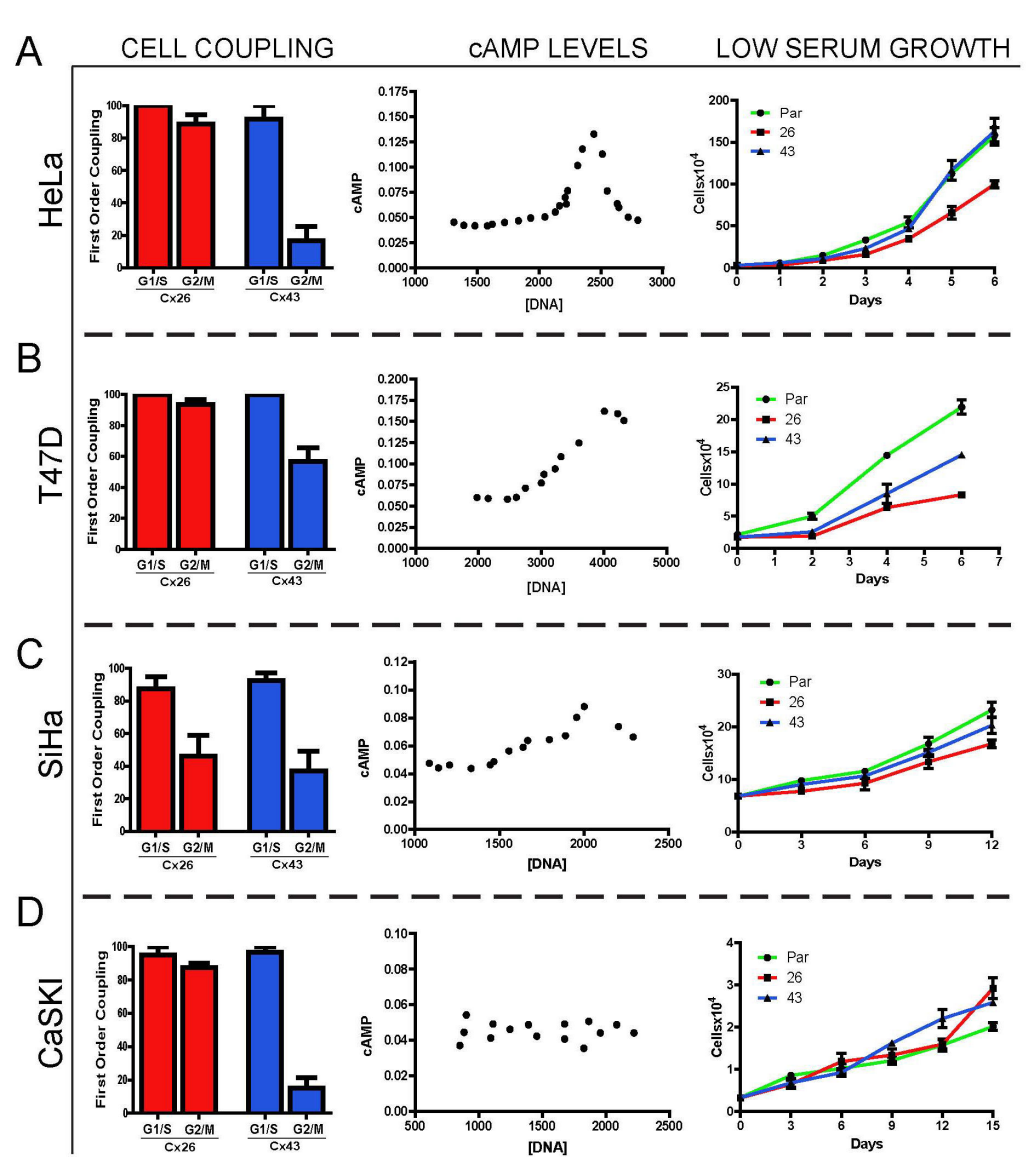
Different cancer cell lines show a predictable inhibition of growth based on cAMP gradients and coupling regulation during the cell cycle. We compared four different breast and cervical tumor cell lines, each of which showed no significant endogenous connexin expression, for: cell cycle regulation of functional coupling by exogenously expressed Cx43 and 26 during the cell cycle (left column); changes in cAMP levels with the cell cycle (middle column), and; growth in low serum of the HeLa Par and Cx43 and 26 transfected cells (right column). All cell lines showed partial loss of Cx43 coupling during M-phase, although the degree of uncoupling varied (T47D showing the least uncoupling), while Cx26 coupling was reduced during mitosis only in SiHa cells. Three of the four cell types show oscillations of cAMP (as measured by FRET as described in Figure 5) throughout the cell cycle, peaking in early M phase, the only exception being CaSKI cells. In all cell types showing cAMP oscillations, connexins which remained coupled during mitosis served as growth inhibitors, with the level of inhibition being correlated with the degree to which they remain open in G2/M. Cell synchronizations, measures of first order coupling and cAMP levels, and growth curves were conducted for each cell line as described for HeLa cells above, although in some cases the duration of the growth curves were extended for tumor cell lines that showed slower growth.

 To test the generality of this mechanism of growth suppression, we sought to determine if additional cell lines that show both cyclical variations in cAMP throughout the cell cycle, and express constitutively functional connexins, would show growth inhibition. Of three additional cancer cell lines tested, including two other cervical cancer lines (SiHa and CaSKI) and a breast cancer cell line (T47D), two (T47D and SiHa) show variations of cAMP with the cell cycle similar to that in HeLa cells (Figure 7). When transfected with Cx26, T47D cells remained coupled throughout the cell cycle, while Cx43 transfectants closed only partially during M phase (Figure 7 row 2). SiHa cells, in contrast, show partial uncoupling of both Cx26 and 43 channels in M phase (Figure 7 row 3). As in HeLa cells (Figure 7 row 1), Cx26 induced growth suppression in both of these cell lines, although this was less marked in SiHa cells where these channels partially uncouple during M phase. Unlike HeLa cells, Cx43 also induced partial growth suppression of both cell types, presumably because it remained partially coupled during mitosis (Figure 7, second row). CaSKI cells showed no cAMP oscillations with the cell cycle, so that even with a similar pattern of cell cycle regulation of Cx43 and 26 coupling as seen in HeLa cells, no growth suppression was evident with either connexin (Figure 7 row 4). Thus, while not all cell types show the same growth response to connexin expression, the effects can readily be explained due to a combination of cAMP variation, and regulation of gap junctional coupling throughout the cell cycle. 

## Discussion

The earliest observation implicating connexins in tumor suppression was made in [6] in 1966. They went on to propose a model of tumor suppression by connexins, in which exchange of metabolites between the non-transformed and transformed cells could suppress growth of the latter by one of two methods: diffusion, and subsequent dilution of mitogenic signal(s), or, receipt of inhibitory signal(s), from adjacent cells. For over forty years, this model has remained hypothetical in nature, due to the failure to identify the molecular signals involved, and the specific pathways they could affect. This has been confounded by the fact that in different types of cancers, different connexins have been reported as effective in growth suppression. Furthermore, functions other than their role as intercellular channels have also been ascribed to connexins, including binding of their carboxy-terminal domain to various growth regulatory proteins [21,22,41], and their ability to form hemichannels on unopposed cell surfaces, which can regulate cell survival [42] or death [19].

 In order to gain a better understanding of the mechanism of growth control, we employed HeLa cells as a broadly studied transformed cell line, which lacks endogenous connexins, but allows for their robust expression exogenously. Consistent with previous findings [14,43], we found that growth of HeLa cells in either low serum, or under anchorage independent conditions, was suppressed ONLY by Cx26, and not by Cx32 or Cx43. These authors suggested that tumor suppression by Cx26 may be independent of gap junction activity [44], based on the observation that two Cx26 mutants, when co-expressed with wild-type Cx26, could reverse growth suppression, even though intercellular communication was maintained. However, communication was only measured qualitatively with synthetic dyes and the effects of multiple transfections were not assessed. Thus, we have applied a more direct strategy to assess the properties of Cx26 responsible for growth suppression, by expressing connexin point mutants that selectively ablate specific connexin functions. Cx26T135A shows complete lack of channel activity, although this mutant forms stable gap junction plaques, indistinguishable from wild type at the electron microscope level [32]. Cx26R75Y forms functional hemichannels, which show only modest gating differences compared to wild type channels [45], but fails to form functional gap junction channels. With the exception of a minor effect on saturation density by Cx26R75Y, neither mutant affected growth rate in low serum or in anchorage independent growth assays. Thus, growth suppression in both assays required intercellular coupling provided by Cx26.

There are several common elements to the growth suppression by gap junctions reported here, and that seen in other cells. Sustained activation of the mitogenic kinases p38MAPK and JNK2 cause G1 arrest in several cell lines [35,46], including HeLa [36]. Similarly, while transient activation of Erk promotes proliferation, sustained activity causes G2/M arrest in varied cell types [47]. In at least one case, this occurred through elevation of p21 levels [48], which we also see here in response to Cx26 coupling. In HeLa cells, specifically, Rahmouni et al. [36], showed that sustained activity of JNK and Erk result in decreased proliferation through blocks in G1 and G2, respectively. The more downstream effectors of cell cycle progression, p21 and p27, have also been associated with Cx43 growth suppression in glioma, osteosarcoma, and kidney tumor cell lines [22,23], while Cx32 deficient mice show decreased levels of p27, and higher incidences of hepatic tumor formation [49]. 

The critical question that has not been addressed in previous studies is how intercellular exchanges through gap junctions can regulate these mitogenic pathways. The first clue in this case was provided by the observed increase in P-PKA, which has been implicated in the activation of most of the mitogenic signaling components identified here, including MEK, Erk [50], p38MAPK [33], and even the levels of p27 [51]. In this system, PKA is the likely regulator of all of these pathways since it was shown to be both necessary and sufficient for Cx26 growth suppression by partial knock-down, and constitutively active expression, of the PKA catalytic subunit, respectively (Figure 4). The second clue came from the finding that PKA activation in HeLa26 cells, as reflected by phophorylation of Thr197, was not associated with increased global cAMP levels. Instead, it was associated with the dissipation of local accumulations of cAMP during M phase of the mitotic cycle, causing a general increase in cAMP levels in the majority of cells in other phases of the cell cycle like G1 and S, resulting in activation of PKA in more cells. This dissipation of cAMP gradients and its dependence on functional intercellular channels that we document here, is consistent with previous demonstrations that cAMP passes effectively through Cx26 channels in HeLa cells [37,38]. Dissipation of cAMP gradients through hemichannels does not appear to play any role, as cAMP gradients are preserved in HeLa Cx26R75Y cells, which actually form more hemichannels than w.t. ([Table pone-0082335-t001]). This is not surprising, as hemichannels are tightly regulated and would be predominantly closed under most physiological conditions. 

**Figure 8:**
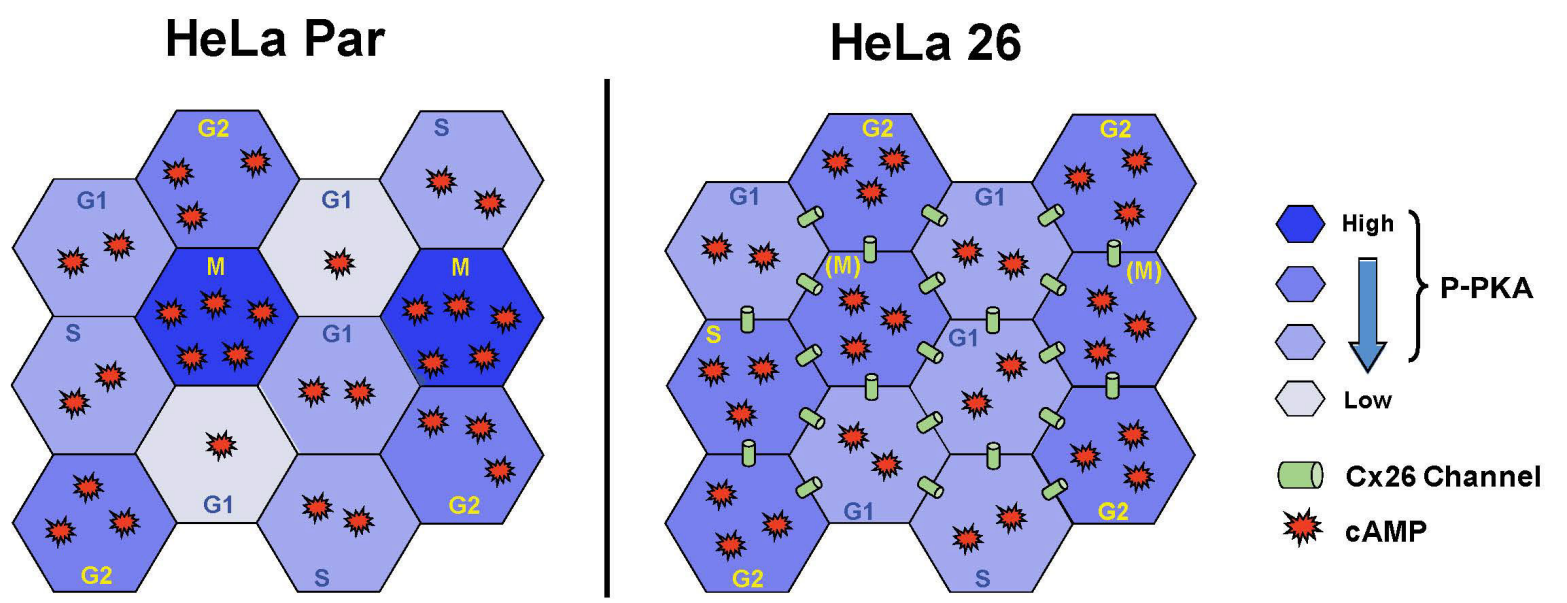
Spatial distribution of cAMP, and effects on PKA activation suggests a model of growth inhibition mediated by gap junctional coupling of cells. cAMP levels oscillate through the cell cycle, as low levels of PKA activity are required during G1 and S phases to allow for progression through interphase, while increasing PKA activity is required to enter mitosis, before lower levels are re-established to promote mitotic exit (see text). In the absence of coupling (left panel), these distinct cAMP (red stars) and P-PKA levels (blue) are maintained as appropriate for each cell cycle phase, even in asynchronously dividing populations. However, a cell population which remains coupled throughout the cell cycle, such as HeLa26 (right panel), will distribute the cAMP more evenly, resulting in dilution of cAMP (and drops in P-PKA) from M phase cells, (resulting in the inhibition of mitotic progression), and concomitant increases in cAMP and PKA activity in G1/S phase cells (resulting in partial G1 arrest).

Activated PKA levels have been shown to oscillate during the cell cycle of many cells, with PKA activity increasing during G2/M, and then decreasing in the latter half of mitosis [52,53], consistent with our results for cAMP levels (Figure 5 E), and those reported earlier in HeLa cells [54]. PKA activity needs to remain suppressed during G1 and S phases, as demonstrated in several studies where addition of exogenous cAMP causes cell arrest in G1/S [55], associated with activation of several cAMP response elements (CRE) that inhibit various steps in S phase [39]. PKA activity then increases in G2/M. where it is required for centrosome duplication [56], and inhibition of mitosis promoting factor (MPF) in order to complete progression through mitosis [57]. The subsequent drop in PKA activity during telophase allows complete exit from mitosis by releasing PKA inhibition of anaphase promoting complex (APC) [58,59]. Thus, as summarized graphically in Figure 8, in HeLaPar cells cAMP levels rise during the early phases of mitosis in order to activate PKA, so P-PKA levels are contributed only by the relatively small number of cells in M phase. However, when the cells are coupled by Cx26, cAMP diffuses out of the M phase cells and into surrounding cells in G1 or S phases. This is sufficient to activate PKA in these cells, resulting in an overall increase in P-PKA in the culture, due to its activation in many more cells. Based on the studies mentioned above, this increase in PKA activity in G1/S phase in HeLa26 cells would be expected to cause significant delays in progression of cells through interphase. The cAMP redistribution would also cause levels to drop during M-phase, possibly causing partial loss of PKA activation that would also produce delays in M phase. 

The redistribution of cAMP can also explain the observed accumulation of multi-lobed nuclei and multi-nucleated cells, and the aberrant appearance of the centrosomes in Cx26 cells (Figure 2). PKA is known to associate with centrosomes up until metaphase [56,60], and to be critical to triggering the separation of centrioles [61]. Localization to centrosomes has been attributed to anchoring of the RIIα subunits to centrosome associated AKAPs [56]. Thus, elevation of cAMP at inappropriate times in the cell cycle would disrupt this association, resulting in failure of, or premature, centriole separation. Consistent with this, we found the phosphorylated form of PKA was often concentrated around the centrosomal area in HeLa Par and Cx43 expressing cells, but this association is less evident in HeLaCx26, which show aberrant centriole duplication patterns.

Cx32 and 43 gap junction channels would be ineffective in redistributing cAMP, as these junctions uncouple at G2/M, isolating those cells with high levels of cAMP. Cell cycle associated regulation of Cx43 has been reported previously and is associated with changes in phosphorylation of Cx43 [49,62] by both PKC [63] and cdc2 [64,65] and internalization of the gap junctions [62,65,66]. We now show that Cx32 channels also uncouple during the cell cycle, but that Cx26 channels do not, showing only a minor drop in coupling during M phase cells and minimal redistribution of protein to the cytosol. 

As the C-terminal domain of Cx43 (or Cx32) contain most of the known sites of kinase action (reviewed in 67), or binding sites for cytoskeletal elements that could affect internalization (reviewed in 26), it is not surprising that its removal causes these connexins to remain open throughout mitosis, like Cx26. This also results in the acquisition of growth suppressive ability of these truncated forms, indistinguishable from that displayed by Cx26. This is consistent with the prior observations in [43]. 

The mechanism for growth regulation proposed above would seem likely to have general applicability, and indeed, in comparisons of several other cancer cell lines, we did find some level of generalization. In three of four cell lines tested, Cx26 did induce growth suppression, although not always to a significantly greater degree than Cx43 (Figure 7). In the one case where no growth suppression was observed (CaSKI cells, Figure 7D), we found that even uncoupled cells did not show any oscillation of cAMP through the cell cycle. Furthermore, in cases where Cx26 and Cx43 showed less difference in their ability to suppress growth, the differences in their regulation during the cell cycle were also much less [either due to Cx43 being less regulated (Figure 7B), or Cx26 showing greater regulation (Figure 7C)]. So while the effectiveness of connexins in growth suppression does vary between cells, this appears to be primarily due to variations in their functionality, or the regulation of cAMP levels, with the cell cycle. 

Overall, this data and the resulting model, provides a unique way of understanding how cell division in a community can be regulated. Rather than overall changes in levels of signaling molecules initiated by an exogenous stimulus, we demonstrate a novel regulatory mechanism based on the spatial distribution of signals within the cell population through gap junctions. While there are likely to be specialized variations on this theme in different cell types, the general principle appears to have wide-spread relevance, and explain why connexins have been associated with tumor suppression in many systems. It may also contribute to our understanding of the association between transient uncoupling of cells and synchronized mitotic responses that occur following growth factor addition [28,63] or partial hepatectomy [30]. The proposed model brings us full circle to the generic models presented by Lowenstein several decades ago, except that we now have finally identified the likely junctional permeant, the signaling pathways, and the redistribution mechanism that can lead to growth inhibition.

## Materials and Methods

### Cells and Transfections/Transductions

Parental HeLa (HeLa Par) cells were a kind gift of Dr. Klaus Willecke. HeLa cells expressing rat Cx26, Cx32, as well as HeLa26R75Y were generated by stable transfection with the pIRESpuro3 vector (Clontech, Palo Alto, CA). They were selected with 1 µg/ml puromycin (Sigma, St. Louis, MO) and maintained in 0.5 µg/ml puromycin. The pIREShygro3 vector was also used to generate Cx26 transfectants as well as Cx26T135A, which were selected in 400 µg/ml hygromycin B (Invitrogen, Carlsbad, CA) and maintained in 200 µg/ml. Stable Cx43 transfectants were generated with the pIRESNeo3 vector and maintained with 1 mg/ml G418 after selection with 2 mg/ml (Invitrogen). All transfections utilized Lipofectamine 2000 with Plus Reagent (Invitrogen). Clonal selection and amplification was performed using Bel-Art Medium Cloning Cylinders (Bel-Art Products, Pequannock, NJ). Several other connexin expressing HeLa cell lines were generated by lentiviral transduction. The lentivirus transfer vector (pSDM-GFP 101), containing the relevant connexin coding region and GFP reporter as a different transcript, was packaged using the envelope (pMD2.G) and packaging plasmid (psPAX2) co-transfected with Lipofectamine LTX with Plus reagent (Invitrogen) into HEK293T cells. Viral supernatant was collected after 48hrs and filtered through a 0.2 µm syringe filter before being added with polybrene (30 µg) to HeLa cells. Second exposure to viral supernatant was used when necessary to achieve >70% infection efficiency as assessed by GFP fluorescence. Comparable studies of the lentiviral transduced cell lines were performed on unselected pools, and this was also performed for the SiHa. CaSKI and T47D cell lines, all obtained from the ATCC. 

### Immunocytochemistry

Cells were plated onto 12 mm round coverslips. At the appropriate time, the cells were fixed with 2% paraformaldehyde (Sigma, St. Louis, MO) for 20 min and processed for immunostaining. 1% BSA (Sigma) was used as the blocking agent. The primary antibodies against, Cx26 andCx32 (Zymed, San Francisco, CA), Cx43 (Santa Cruz Biotechnology, Santa Cruz, CA), γ-tubulin (Pierce, Rockford, IL) and T197 phospho-catalytic subunit of PKA (Cell Signaling Technology, Danvers, MA) at 1:50 dilution) were incubated for 1 hr at room temperature, all at 1:100 dilution. The appropriate secondary antibodies conjugated to Alexa 488 or Alexa 594 (Molecular probes, Invitrogen) were used at 1:200 concentration for 45 min at room temperature. Washes were performed with Phosphate Buffered Saline (PBS) with 1% Tween (Sigma). 4',6-diamidino-2-phenylindole (DAPI) (Sigma) or Hoechst33342 (Sigma) was used to counterstain the nucleus. The coverslips were mounted on glass slides using Prolong Gold Mounting Reagent with Antifade (Invitrogen), and the edges were sealed with clear nail polish before imaging. Images of connexins were collected using a 60X PlanApoN NA 1.42 oil immersion objective on an Olympus IX-81 confocal microscope (Olympus, Center Valley, PA) equipped with an Olympus Fluoview 500 laser scanning system and a photomultiplier tube. Alexa-488 was excited by the 488 nm line of an Argon laser and imaged through a 505-525 nm band pass filter. Hoechst33342 was excited by the 405 nm line of a blue diode laser and imaged through a 430-460 nm band pass filter. P-PKA was imaged using a Nikon 40X PlanFluor NA 1.3 oil immersion objective on a Nikon TE-2000U (Nikon, Melville, NY) inverted epifluorescent microscope equipped with a Prior Lumen 200 light source (Prior Scientific, Rockland, MA) and a Photometrics CoolSnap HQ^2^ quantitative CCD camera (Photometrics, Tucson, AZ). Alexa 488 and Alexa 594 were imaged through Nikon B-2E/C and Y-2E/C filter sets, respectively, and DAPI was observed with a UV-1A filter set. All final images are compiled projections of a confocal Z-series collected at 0.5 mm steps with Olympus FluoView software, except those for P-PKA which represent the best focal planes and were analyzed with Metamorph Software (Molecular Devices, Downingtown, PA). . 

### Hoechst and Annexin-V staining

The staining kits for Hoechst, Annexin-V, and TUNEL apoptotic assays were purchased from Invitrogen. Cells were trypsinized, pooled with floating cells, washed, and resuspended in PBS. The cells were then stained with the appropriate dyes according to manufacturer’s instructions. The raw data for Annexin-V with propidium iodide and TUNEL assays was collected by FACSCalibur (BD Biosciences, San Jose, CA). Hoechst with propidium iodide staining was collected using FACSAria (BD Biosciences). Data analyses were performed by Cell Quest software (BD Biosciences) for all the assays.

### Dye transfer and dye uptake assay

Coupling of connexin transfected cells was assayed using the pre-loading method of calcein dye transfer [68]. In order to asses GJIC during the cell cycle, synchronized cells in DMEM with 10% FBS in 35mm^2^ dishes were injected with the gap junction permeable fluorescent dye Alexa 488 (FITC (Invitrogen) by using an Eppendorf Femtojet microinjector (Eppendorf, Hauppauge, NY). Images were acquired with a Nikon TE-2000U microscope using a Nikon 20X ELWD PlanFluor .45NA objective and a 1.5x multiplier. All injections were performed at 37°C in a temperature controlled environment. Image acquisition and analysis was done through Metamorph software that was set up to automatically take an image 1 minute after injection, which was selected as the cutoff point as most transfer is very rapid and occurs in the first minute after injection. Coupling was analyzed both with respect to first order neighbors as well as the total number of cells filled based on fluorescent image overlays with transmitted light images of the same fields. At least 10 cells of each connexin type were injected at each time point to be used for statistical comparisons. 

 To assay hemichannel function, modification of a previously described protocol [69] was used. Cells were grown on 24-well plates, washed two times with 1 ml PBS, and mechanically stimulated by adding 0.5 ml of PBS drop-wise using a 1 ml pipetman in all the four quadrants of the well. Immediately, 2% Lucifer yellow and 2% Rhodamine dextran (Molecular Probes) were added and incubated for 10 min in PBS. The latter dye is hemichannel impermeable and used to mark cells with leaky membranes. The cells were washed 5-7 times and cells taking up Lucifer Yellow, but not Rhodamine dextran, were counted at room temperature using a Zeiss Axiovert35 photomicroscope equipped with UV epifluorescence and a 10X Zeiss PlanNEOFUAR .3 NA objective. Data was recorded on an AxioCamMR CCD camera and stored digitally using AxioVision software (Carl Zeiss, Germany).

### Cell growth rates in low serum condition

HeLa Par and transfected cells were seeded at 3.3x10^4^ cells/well in 24-well plates with DMEM culture medium containing 10% fetal bovine serum (FBS) (Invitrogen). Individual clones of connexin transfected cells were mixed in equal ratios prior to each experiment. The medium was removed after 16 hours, cells were rinsed with PBS, and DMEM without added serum. After three days of serum starvation, DMEM with 1% FBS was added and replaced every 24 hr. Cells were seeded in triplicate in all experiments and at each time point (approximately every 24 hr), triplicates were treated with 0.25% trypsin (Invitrogen) and collected for counting using a hemoctyometer.

### Cell Synchronization

For G1/S arrested populations, HeLa cells plated at 20% confluency in DMEM (Invitrogen, Carlsbad, CA) with 10% FBS were incubated successively with 2 mM thymidine (Sigma) for 18 hours, without thymidine for 9 hours, and again in 2 mM thymidine for 17 hours. After release from thymidine block, progression through the cell cycle was monitored by FACS analysis. For G2 arrested populations, HeLa cells were plated at 30% confluency and incubated for 24 hours with 2mm thymidine, released for three hours in fresh medium, and incubated for 12 hours with 100 ng/ml Nocodazole (Enzo Life Sciences, Farmingdale, NY). After release from Nocodazole block, progression through the cell cycle was again monitored by FACS analysis. 

### Cell cycle analysis

Cells were trypsinized, washed in PBS, and fixed with chilled 100% ethanol for 15min at -20°C, before resuspension in SSC buffer (15mM Sodium citrate, 150mM Sodium chloride, pH 7.0). Next, they were treated with 0.1 mg/ml RNaseA (Sigma) in 0.1 % TritonX-100 (Acros Organics, Morris Plains, NJ) for 30 min at 37°C. After removal of RNaseA, cells were resuspended in SSC buffer and 0.01 mg/ml propidium iodide (Sigma) was added. After incubation for 10 min at RT, the raw data was collected by FACSCalibur (BD Biosciences) using 488nm and 617nm (excitation and emission wavelengths respectively), detected in FL2 and FL3 channels and analyzed by ModFit (Topsham, Maine) or Flow Jo (Treestar, Ashland, OR) software.

### Western blots

For detection of connexin proteins, total cell lysates were prepared by adding 1 ml RIPA buffer (PBS, 1% NP40, 0.5% sodium deoxycholate, 0.1% SDS, 10 mg/ml PMSF, 10 μg/ml Na3VO4, aprotinin, leupeptin, pepstatin) to 100 mm^2^ dishes and scraping with cell scrapers. Lysates were passed through a needle 18 times to shear the DNA and spun at 15,000 rpm for 20 minutes. Protein estimations were performed on the supernatant using the BioRad DC protein assay kit (BioRad, Hercules, CA) with BSA (Sigma) as a standard. 5 μg of protein per sample were separated on a 12% SDS gel and electroblotted onto ImmobilonP membranes (Millipore, Bedford, MA). The blots were probed with monoclonal anti-Cx43 antibody at 1:1000 dilution (BD Biosciences), monoclonal anti-Cx32 antibody at 1:500 dilution (Zymed, San Francisco, CA), and monoclonal anti-actin antibody at 1:2000 dilution (Sigma). For detection of kinase activity, total cell lysates were prepared by pooling both the attached and floating cells and directly boiling them in SDS loading buffer (6X concentration: 250 mM TrisHCl pH 6.8, 10% SDS, 40% glycerol). The lysates were kept on ice for 10 min, and then centrifuged at 15,000 rpm for 20 minutes to remove insoluble components. Protein estimations were performed on the supernatant using the MicroBCA protein assay kit (Pierce, Rockford, IL). 100 mM DTT and 0.01% bromophenol blue was added to 10 μg of protein per sample, which were separated on an SDS 12% polyacrylamide gel and electroblotted onto ImmobilonP membranes (Millipore). The blots were probed with the appropriate antibodies. All of the kinase and cell cycle antibodies were purchased from Cell Signaling Technology (Danvers, MA) and used at their recommended concentrations and conditions. 

Actin antibody (Sigma, St. Louis. MO) was used at a dilution of 1:100,000. The chemi-luminiscent femto-sensitive developing reagent (Pierce, Rockford, IL) was used to develop images for visualization by X-Ray films (Kodak *RP* X-OMAT Processor, Model M6B). Images were also captured using G:Box from Syngene Bioimaging systems (Frederick, MD) and quantitated using the manufacturer’s Genetools software. The blots were stripped (100 mM 2-mercaptoethanol, 2% (w/v) SDS, 62.4 mM Tris-HCl, pH 6.7) for 30 min at 50°C with mild agitation and re-probed with antibodies against total kinase, followed by actin to serve as loading controls. 

### Distribution of cellular P-PKA

Cells were probed with an antibody to the T197 phosphorylated catalytic subunit of PKA (Cell Signaling Technology, Danvers, MA), followed by a secondary antibody bound to Alexa 488 (Molecular probes), as well as a DAPI nuclear stain. Epifluorescent images were taken as described above. The Cell Scoring Application Module in the MetaMorph software (Molecular Devices) was used for cellular segmentation and to quantify the pixel intensity of P-PKA staining for the cytoplasm and the nucleus. Because the staining was almost exclusively cytoplasmic, histograms of the cytoplasmic pixel intensities versus DAPI staining for each cell were generated in SigmaPlot (Systat Software, San Jose, CA). 

### cAMP measurement

cAMP assays were performed with the cAMP EIA kit from Cayman Chemical (Ann Arbor, MI) with minor modifications. Briefly, cells in three separate 35mm^2^ dishes per connexin type per day were each incubated in ice cold 5% TCA for 2min with swirling to immediately stop kinase and phosphatase activity. The mixtures were then individually collected, centrifuged at 1500g in a microfuge, and the supernatants ether extracted and lyophilized. The dried pellets were resuspended in EIA buffer and used directly in the assay. All raw cAMP concentrations were then normalized to the average protein levels from three additional 35mm^2^ dishes per connexin type that were not used in the assay. 

### Statistical Analyses

The Student’s t-test was used to determine the significance level of difference between pairs of data sets. P<0.05 or lower was considered significant. For multiple data sets, multivariate one-way ANOVA was performed followed by non-parametric Bonferroni or Tukey post-hoc tests at a 95% confidence interval with Graphpad Prism Software (Graphpad Software, La Jolla, CA).

### cAMP FRET Analysis

The cAMP sensor H84 was created and characterized by Kees Jalink (van der Krogt et al., 2008). It is expressed from a pCDNA3 vector (Invitrogen) and includes a cAMP binding domain from Epac, a cAMP-regulated guanine nucleotide exchange factor, which is between CFP donor and Venus (YFP) acceptor fluorophores. H84 transfection was carried out with Lipofectamine as described previously in HeLa cells plated on 12 mm^2^ #1 thickness coverslips (Fisher Scientific, Pittsburgh, PA). The coverslips were fixed for 20 min in 3.7% paraformaldehyde (Sigma) in PBS and washed 3 times for 5 minutes in PBS. Acceptor photobleaching FRET imaging was performed immediately after washing using a 12mm^2^ quick change coverlip chamber (Warner Instrument Company). Images were obtained with a 63X Plan-ApoCHROMAT 1.40 NA oil immersion objective on a Zeiss 510 NLO confocal inverted microscope (Carl Zeiss, Germany) equipped with a photomultiplier tube and analyzed with Zeiss LSM Imaging Software. The CFP and Venus fluorophores were excited with an Argon laser at 458 nm (10% power) and 514 nm (3% power), respectively. CFP emission was detected with a 470-500 nm bandpass filter and YFP emission was detected with a 525-575 nm band pass filter. After the initial image for each fluorophore was acquired, the YFP acceptor was bleached in the entire cell at 100% power, and images were again immediately collected for each channel. For FRET values, regions were drawn encompassing the entire surface area of each cell and the pixel intensity values of the fluorophors were averaged from all the values within the region before and after bleaching. The FRET percent equation utilized was: 

*[(CFP post – CFP pre*)*/CFPpost*]*/[(YFPpre – YFPpos*)*/YFPpre*]* X 100*

The FRET efficiency equation used was:

1-*(YFPpre/YFPpost*)

CFP alone, YFP alone, and co-transfected CFP with YFP were used as controls for spectral overlap and bleedthrough, which were found to be insignificant. For cAMP FRET specificity, H84 transfected cells were incubated with 100µM each of 8-Br-cAMP (Sigma), IBMX (Enzo Lifesciences), and Forskolin (Enzo Lifesciences). The objective, laser power, pinhole diameter, and bleach times were kept constant for all experiments. 

## Supporting Information

Figure S1 **Connexin expression among the different clones (related to Figure 1).** HeLa clones stably transfected with different connexins were tested for expression by Western blot. Left panel - Cx43 antibody, middle panel - Cx32 antibody, and right panel - Cx26 antibody (P: parental (untransfected) cells; wt: wild type Cx26, R: Cx26R75Y, T: Cx26T135A). Bottom panel shows corresponding actin staining as a loading control.
(TIF)

Figure S2 **Growth of different HeLa clones in anchorage independent conditions (related to Figure 1).** Growth comparison of HeLa Par (open bars) with pools of HeLa clones transfected with Cx43 (closed bars), Cx32 (horizontally lined bars), Cx26 (double cross-hatched bars), Cx26R75Y (right cross-hatched bars) or Cx26T135A (left cross-hatched bars) subjected to growth under anchorage independent conditions for 6 days. Representative data from one of three experiments is shown, with points representing the mean ± SEM of triplicate platings.
(TIF)

Figure S3 **Representative Western blot showing differential long term kinase activation in HeLa26 compared to HeLa Par cells (related to Figure 3).** Representative Western blots show the differential activation of cdk inhibitors and kinases (indicated to the right of each blot) in HeLa Par (P) and HeLa26 (26), at 0-3 days after 1% serum addition following serum starvation. A ratio of phosphorylated (P-) and total forms of each kinase derived from digital images yield the activation levels that are plotted in Figure 3 of the main manuscript. The arrow indicates the 54 kDa JNK2 isoform which is believed to mediate the anti-proliferative effects of P- JNK. Actin serves as the internal loading control.
(TIF)

Figure S4 **Western blot showing immediate early activation of kinases in HeLa Par and HeLa26 (related to Figure 3).** Immediate early response of the kinases was assessed at 0, 20 min, 45 min, 2h and 8h after 1% serum addition following serum starvation by comparing levels of phophorylated forms (upper panels) and total protein levels (lower panels) for each kinase. In HeLa Par, Erk and JNK show maximum activation at 20-45 min, followed by resumption of the basal state, and longer term activation in the case of JNK. In contrast, HeLa26 shows no peak in activity at these early time points, but only a longer term increase in phosphorylated forms of these kinases beyond 2 hours. p38MAPK shows a dip in activity in HeLa Par at 20 and 45 min, perhaps indicative of the abolition of the anti-proliferative effect of p38MAPK immediately after serum addition. HeLa26 shows no change in activity of p38 over the time course shown.
(TIF)

Table S1 (DOCX)
