# Supplementary figures and images for: Intercellular Redistribution of cAMP Underlies Selective Suppression of Cancer Cell Growth by Connexin26
Source: PLoS One. 2013 Dec 3;8(12):e82335. doi: 10.1371/journal.pone.0082335 (PMC3849486; doi:10.1371/journal.pone.0082335)

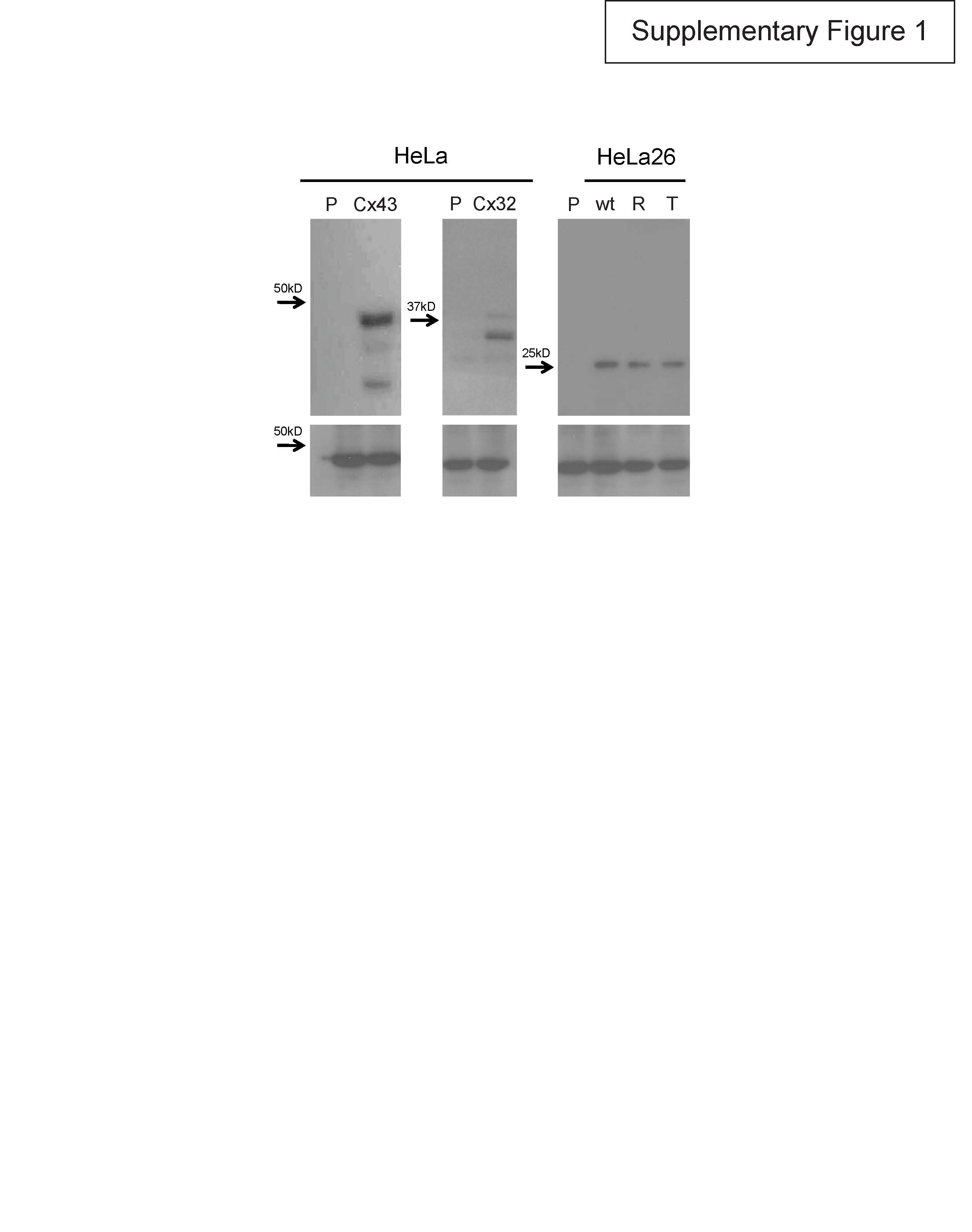

Supplement: Figure S1 — Connexin expression among the different clones (related to Figure 1). HeLa clones stably transfected with different connexins were tested for expression by Western blot. Left panel - Cx43 antibody, middle panel - Cx32 antibody, and right panel - Cx26 antibody (P: parental (untransfected) cells; wt: wild type Cx26, R: Cx26R75Y, T: Cx26T135A). Bottom panel shows corresponding actin staining as a loading control. (TIF) [file pone.0082335.s001.tif]

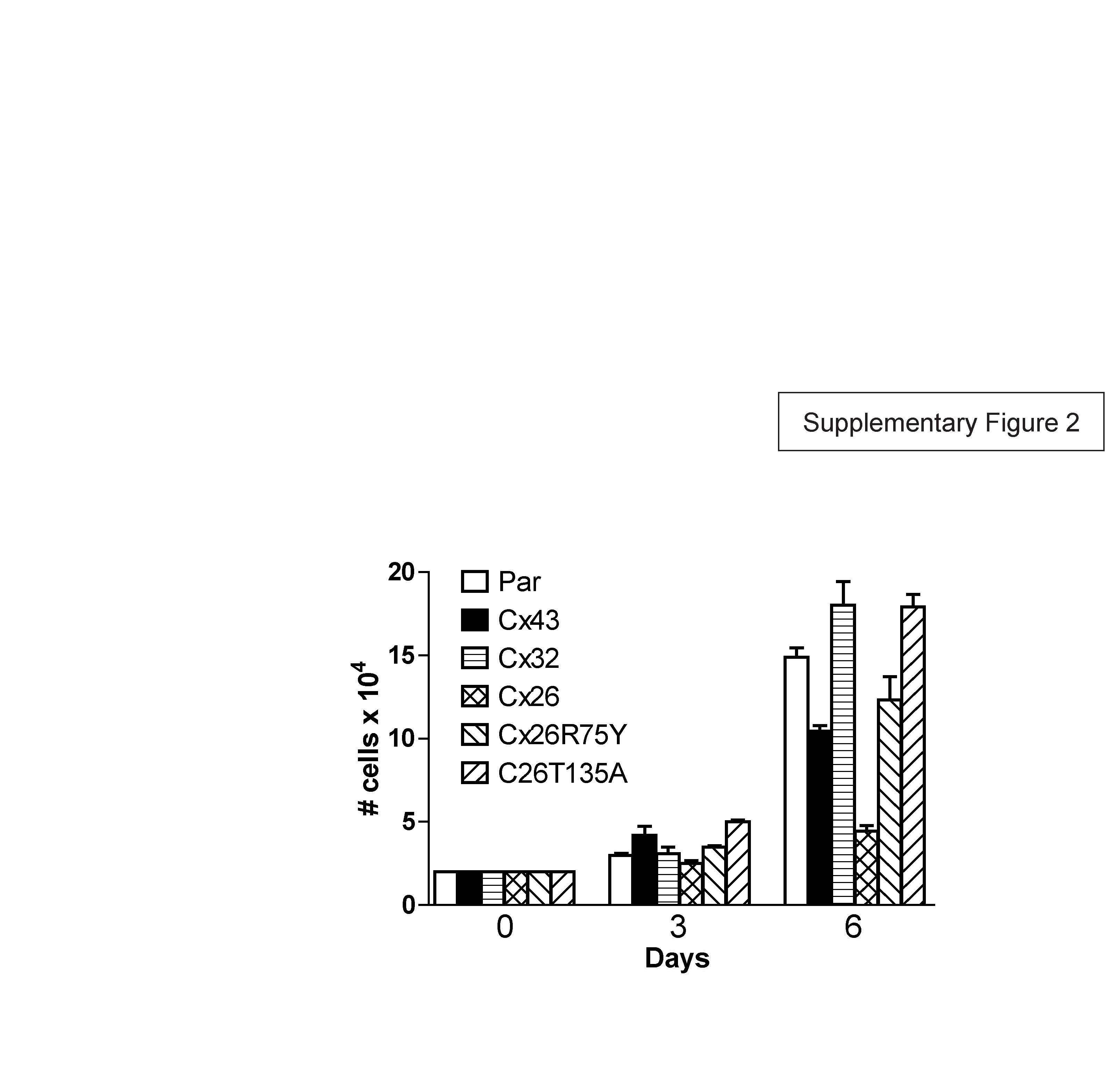

Supplement: Figure S2 — Growth of different HeLa clones in anchorage independent conditions (related to Figure 1). Growth comparison of HeLa Par (open bars) with pools of HeLa clones transfected with Cx43 (closed bars), Cx32 (horizontally lined bars), Cx26 (double cross-hatched bars), Cx26R75Y (right cross-hatched bars) or Cx26T135A (left cross-hatched bars) subjected to growth under anchorage independent conditions for 6 days. Representative data from one of three experiments is shown, with points representing the mean ± SEM of triplicate platings. (TIF) [file pone.0082335.s002.tif]

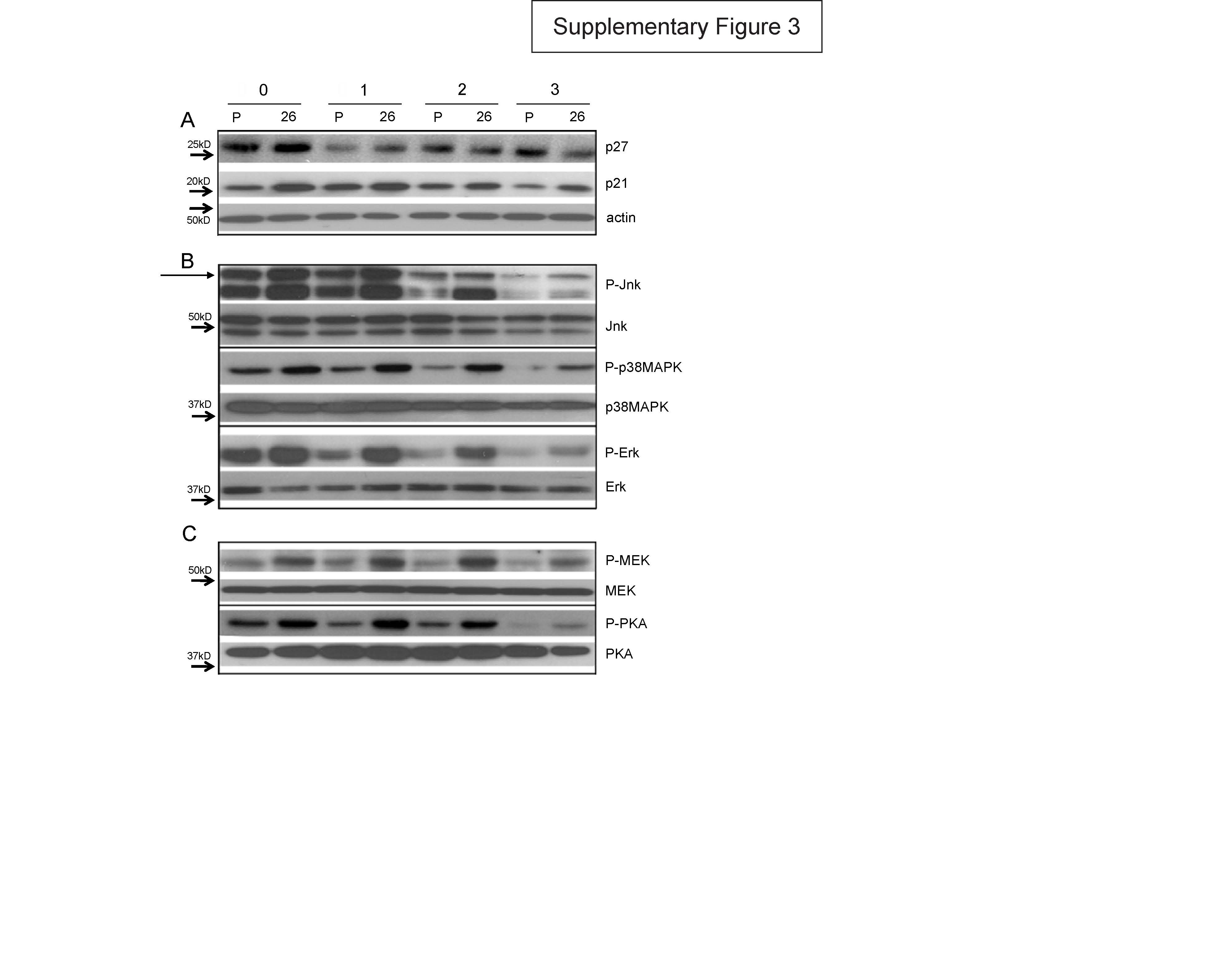

Supplement: Figure S3 — Representative Western blot showing differential long term kinase activation in HeLa26 compared to HeLa Par cells (related to Figure 3). Representative Western blots show the differential activation of cdk inhibitors and kinases (indicated to the right of each blot) in HeLa Par (P) and HeLa26 (26), at 0-3 days after 1% serum addition following serum starvation. A ratio of phosphorylated (P-) and total forms of each kinase derived from digital images yield the activation levels that are plotted in Figure 3 of the main manuscript. The arrow indicates the 54 kDa JNK2 isoform which is believed to mediate the anti-proliferative effects of P- JNK. Actin serves as the internal loading control. (TIF) [file pone.0082335.s003.tif]

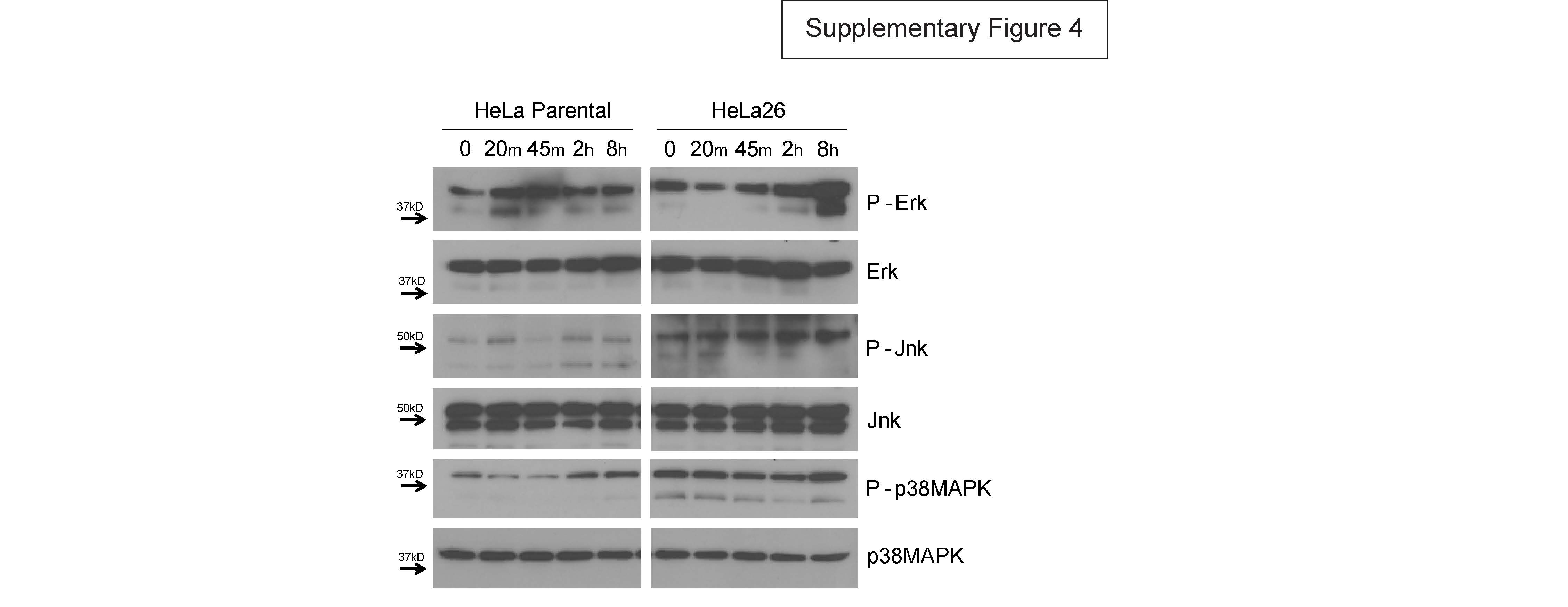

Supplement: Figure S4 — Western blot showing immediate early activation of kinases in HeLa Par and HeLa26 (related to Figure 3). Immediate early response of the kinases was assessed at 0, 20 min, 45 min, 2h and 8h after 1% serum addition following serum starvation by comparing levels of phophorylated forms (upper panels) and total protein levels (lower panels) for each kinase. In HeLa Par, Erk and JNK show maximum activation at 20-45 min, followed by resumption of the basal state, and longer term activation in the case of JNK. In contrast, HeLa26 shows no peak in activity at these early time points, but only a longer term increase in phosphorylated forms of these kinases beyond 2 hours. p38MAPK shows a dip in activity in HeLa Par at 20 and 45 min, perhaps indicative of the abolition of the anti-proliferative effect of p38MAPK immediately after serum addition. HeLa26 shows no change in activity of p38 over the time course shown. (TIF) [file pone.0082335.s004.tif]
