# Supplementary material for: Intercellular Redistribution of cAMP Underlies Selective Suppression of Cancer Cell Growth by Connexin26
Source: PLoS One. 2013 Dec 3;8(12):e82335. doi: 10.1371/journal.pone.0082335 (PMC3849486; doi:10.1371/journal.pone.0082335)
Supplement: Table S1 — (DOCX) [file pone.0082335.s005.docx]

**Table S 1:** Effects of connexin expression on cell viability

| **HeLa Cell Type** | **% Detached Cells** | **% TUNEL Stained Cells** | **Annexin-V Positive Cells** | **% Hoechst Stained Cells** |
| --- | --- | --- | --- | --- |
| **Parental** | 1±0.2 | 13.83 | 14±1 | 9±1 |
| **Cx43** | 1±0.1 | 12.83 | 19±1 | 9 |
| **Cx32** | 2±0.3 | 11.75 | 16±1 | 8±1 |
| **Cx26** | 3±0.4^a^ | 14.03 | 24±1 ^a b^ | 20±1 ^a b^ |
| **Cx26R75Y** | 1±0.1 | n.d. | 17±1 | 10 |
| **Cx26T135A** | 1±0.1 | 3.14 | 14±1 | 9±1 |

^a^ indicates significance elevated at a p<0.05 compared to all other cell types.

^b^ morphological staining revealed these cells to be non-apoptotic, as they did not show condensed or fragmented nuclei (as seen in Figure 3B). These cells were likely blocked in cytokinesis, and thus more “leaky” than other cells, and stained more effectively with Hoechst and Annexin V. They also likely contribute to the higher number of detached cells in HeLa26.

n.d.: not detectable
